# NAC-mediated membrane lipid remodeling negatively regulates fruit cold tolerance

**DOI:** 10.1093/hr/uhac039

**Published:** 2022-05-04

**Authors:** Chunbo Song, Mengbo Wu, Ying Zhou, Zehao Gong, Weiwei Yu, Yi Zhang, Zhenfeng Yang

**Affiliations:** College of Biological and Environmental Sciences, Zhejiang Wanli University, Ningbo, Zhejiang 315100, China; College of Food Science and Engineering, Ocean University of China, Qingdao, Shandong 266100, China; Key Laboratory of Plant Hormones and Development Regulation of Chongqing, School of Life Sciences, Chongqing University, Chongqing 401331, China; State Key Laboratory of Biocontrol, Guangdong Provincial Key Laboratory of Plant Resources, Collaborative Innovation Center of Genetics and Development, School of Life Sciences, Sun Yat-sen University, Guangzhou 510275, China; Key Laboratory of Plant Hormones and Development Regulation of Chongqing, School of Life Sciences, Chongqing University, Chongqing 401331, China; State Key Laboratory of Biocontrol, Guangdong Provincial Key Laboratory of Plant Resources, Collaborative Innovation Center of Genetics and Development, School of Life Sciences, Sun Yat-sen University, Guangzhou 510275, China; State Key Laboratory of Biocontrol, Guangdong Provincial Key Laboratory of Plant Resources, Collaborative Innovation Center of Genetics and Development, School of Life Sciences, Sun Yat-sen University, Guangzhou 510275, China; College of Biological and Environmental Sciences, Zhejiang Wanli University, Ningbo, Zhejiang 315100, China

## Abstract

Low temperatures are known to destroy cell membranes’ structural integrity by affecting the remodeling of their phospholipids. Fruits stored at low temperature are prone to chilling injury, characterized by discoloration, absence of ripening, surface pitting, growth inhibition, flavor loss, decay, and wilting. Phosphatidic acid, a vital second-messenger lipid in plants, is known to accumulate in response to different kinds of stress stimuli. However, the regulatory mechanism of its production from the degradation of phospholipids remains poorly understood. We identified two cold-responsive NAC (NAM/ATAF1/CUC2) transcription factors from bananas, namely, MaNAC25 and MaNAC28, which negatively regulated cold tolerance in banana fruits by upregulating the expression of phospholipid degradation genes in banana fruits. Furthermore, MaNAC25 and MaNAC28 formed a positive feedback loop to induce phospholipid degradation and produce phosphatidic acid. In contrast, ethylene directly inhibited the degradation of phospholipids in banana and transgenic tomato fruits. In addition, ethylene reduced the activity of MaNAC25 and MaNAC28, thereby inhibiting phospholipid degradation. To conclude, NAC-mediated membrane lipid remodeling negatively regulates the cold tolerance of banana and transgenic tomato fruits.

## Introduction

Chilling injury (CI) is caused when cold-sensitive fruits and vegetables are exposed to extremely low temperatures [1] that severely affect their quality and shelf-life. The cold signaling pathway in plants is known to be regulated by numerous cold-regulated (*COR*) genes that are activated by the inducer of C-repeat binding factor (CBF) expression (ICE)-responsive element-binding factor 1 (CBF/DREB1) transcriptional cascade [2]. In addition, increasing evidence suggests a variety of other pathways involved in the induction of cold tolerance in plants [3].

Under cold stress, remodeling of both plastidic and extra-plastidic membrane lipids affects plant cells’ activity and their subsequent survival [4–6]. For example, cold stress significantly changes the levels of unsaturated fatty acids and phospholipids, consequently altering the membrane fluidity and integrity [7]. The structural backbone of biological membranes is constituted by various phospholipids, including phosphoinositide (PI), phosphatidylglycerol (PG), phosphatidylserine (PS), phosphatidylethanolamine (PE), phosphatidylcholine (PC), and phosphatidic acid (PA). Phospholipases, known to hydrolyze phospholipids, have been implicated in plant growth and development also in response to several kinds of stress, comprising cold, pathogen attack, drought, and wounding [8]. Among these phospholipids, PA, a polar lipid without a head group, forms a destabilized hexagonal II (H_II_)-type phase with other lipids [7], thereby directly affecting the stability of the membrane phospholipid bilayer [9]. Its reaction with reactive oxygen species (ROS), which accumulates during stress, is known to promote cell death [1, 10]. Furthermore, PA binds to a series of proteins as part of multiple signal transduction pathways and molecular reactions mediating stress responses [8, 11]. For instance, PA modulates plant potassium (K^+^) channels by inhibiting the function of OsAKT2, thus affecting plant growth and development [12]. Lin *et al*. [13] reported a link between PA and vacuolar degradation of PIN2 in modulating root hair development under phosphorus deficiency [13]. Moreover, Li *et al*. [14] developed a biosensor to determine the dynamic changes in PA concentrations in cell types and diverse tissues in response to stress stimuli. This biosensor revealed an association between PA signaling and cellular pH that determined *Arabidopsis* tolerance to salt stress [14]. Hence, PA, an important second messenger lipid in higher plants, regulates development and signal transduction [8, 15]. PA is generated via two signaling pathways. First, it can be directly produced by the hydrolysis of structural lipids such as PC and PE by phospholipase D (PLD) [16, 17]. Second, hydrolysis of phosphatidylinositols by phospholipase C (PLC) produces diacylglycerol, which is rapidly phosphorylated by diacylglycerol kinase (DGK) to produce PA [15, 18]. PA participates in different stress responses and rapidly accumulates in response to various environmental signals [19–21]. For example, cold stress in *Arabidopsis* suspension cells induces PLC and PLD activity and rapid accumulation of PA [22]. Similarly, *dgk2*, *dgk3*, and *dgk5* knockout mutants improve the tolerance and attenuate PA accumulation in response to freezing temperatures [23]. These findings indicated that PA accumulation is intricately related to cold stress, with PLD, PLC, and DGK as key enzymes responsible for its production. However, literature on the genetic, transcriptional, and post-transcriptional regulation of PA production under cold stress via phospholipid degradation is scarce.

The NAC (NAM/ATAF1/2/CUC2) proteins, characterized by a highly conserved NAC domain, constitute one of the largest plant transcription factor (TF) families. The NAC domain is composed of five subdomains (A–E) in the highly diversified C-terminal region, which serves as the transcription activation domain, and the N-terminal region, which functions as a DNA-binding domain [24]. Moreover, NAC TFs have been implicated in regulating complex signaling networks that function in response to various abiotic stresses, such as hypoxia, drought, cold, and salt. In addition, NAC TFs also regulate hormone signaling in plants and include ethylene, jasmonic acid, gibberellic acid, indole-3-acetic acid, salicylic acid, and abscisic acid (ABA) signaling [25–27]. NAC TFs can act both as activators and repressors of various plant physiological processes. For example, the majority of NACs, such as tomato SlJUB1, SlNAC4, and SlNAC35 [28–30], cotton GhNAC2 [31], maize ZmSNAC1 and ZmNAC33 [32, 33], rice ONAC106, OsNAC6, and ONAC022 [34–36], and banana MusaSNAC1, MusaNAC042, and MpSNAC67 [37–39], serve as positive regulators in the tolerance processes of several abiotic stresses, including dehydration, drought, salt, ABA, and osmotic stresses. Only a few NACs, such as ZmNAC071 [40], tomato SlSRN1 [41], and *Arabidopsis* NAC016 [42] and NTL4 [43], act as negative regulators. In addition, NACs such as tomato SlNAC1 and SlNAM1 [44, 45] and grape VvNAC17 [46] are known to positively regulate cold tolerance in plants. However, negative regulation by NACs in plant cold tolerance has rarely been reported in *Arabidopsis* and fruits. Currently, in the wild tomato species *Solanum habrochaites* a novel NAC TF ShNAC1 has been reported to negatively regulate cold tolerance in transgenic tomato plants [47]. Similarly, CaNAC1 TF is known to regulate PA production in green bell pepper in response to cold stress [48]. However, a detailed regulatory mechanism in fruits in response to cold stress remains elusive. Thus, there is an urgent need to characterize the functions of cold-responsive NAC TFs in regulating phospholipid degradation to produce PA and to improve understanding of plant responses to cold stress.

Banana is highly sensitive to CI when stored at temperatures below 13°C. CI in the banana fruit is characterized by peel browning, flesh mealiness, abnormal ripening, and increased decay; this is the major limiting factor for refrigerated preservation of bananas [49]. Therefore, understanding the physiological response of bananas to cold stress and improving techniques to induce cold tolerance of harvested banana fruits are necessary for their safe storage and transportation. Application of several exogenous substances, such as jasmonic acid, ABA, progesterone, 24-epibrassinolide, nitric oxide, fibroin, and ethylene, can enhance cold tolerance in banana fruits [50–58]. Previous research has reported that propylene, an ethylene analog, induces cold tolerance in bananas via MaNAC1-mediated regulation of the ICE1-CBF cold signaling pathway [27]. However, whether ethylene regulates NAC TFs through other metabolic pathways, such as the phospholipid degradation pathway, to improve the cold tolerance of banana fruits is largely unknown.

**Figure 1:**
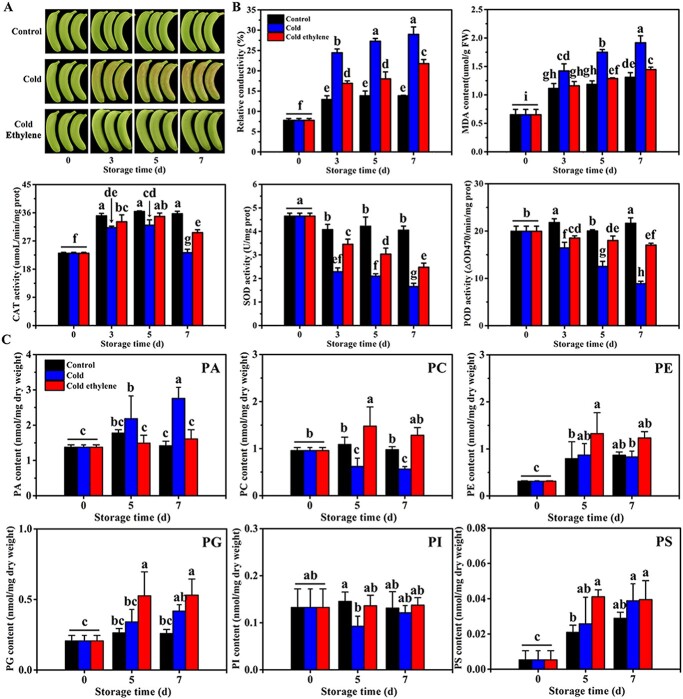
Apparent traits and changes in physiological indexes and total lipid content of banana fruits in control (22°C), cold (7°C), and cold ethylene (pretreatment with 500 ppm ethephon and then transfer to 7°C) treatment groups. **a** CI phenotype of banana fruit in the control, cold, and cold ethylene treatment groups. **b** Relative conductivity, MDA content and CAT, SOD, and POD activity assays in banana fruit in the control, cold, and cold ethylene treatment groups. Error bars show the standard error from three replicates. Statistical analysis was performed using ANOVA followed by Duncan’s multiple range test at *P* = .05. **c** Lipid content of banana fruit under three sets of treatments; normal temperature (22°C) was used as control. Values are mean ± standard error (nmol/mg dry weight; *n* = 4). Significant differences among the three groups were determined by ANOVA followed by Duncan’s multiple range test at *P* = .05.

In this research, we identified two cold-responsive NAC TFs from bananas, namely, MaNAC25 and MaNAC28, which belong to two different subgroups within the NAC family. MaNAC25 and MaNAC28 have been reported to negatively regulate cold tolerance in banana fruits by activating the transcription of phospholipid degradation genes (*MaPLDα1*/*4*, *MaPLDβ1*/*2*/*3*, *MaPLDδ1*/*2*/*5*, *MaDGK3*, and *MaPLC1*/*2*), and forming a positive feedback loop to induce PA accumulation. We demonstrated that ethylene reduced the activity of MaNAC25 and MaNAC28, thereby inhibiting phospholipid degradation to produce PA in banana and transgenic tomato fruits, further confirming that MaNAC25 and MaNAC28 negatively regulate fruit cold tolerance. Altogether, our results suggest a lipid metabolism-based regulatory mechanism by which NAC TFs negatively regulate cold tolerance in banana and transgenic tomato fruits.

## Results

### Ethylene enhances cold tolerance in banana fruits

Ethylene effectively improved the cold tolerance of banana fruits (Fig. 1a), the same finding as previously reported [27, 52]. Furthermore, malondialdehyde (MDA) content and relative conductivity in cold-treated fruits increased with increasing storage time at 7°C. These values were significantly higher than those in the control group bananas stored at 22°C (Fig. 1b). Next, the activities of catalase (CAT), superoxide dismutase (SOD), and peroxidase (POD), which are ROS-scavenging enzymes, were examined. As shown in Fig. 1b, CAT activity first increased, followed by a decrease, whereas SOD and POD activities displayed a downward trend in fruits stored at 22 and 7°C. Compared with the control group, the activities of CAT, SOD, and POD were significantly decreased in the cold-treated group during the storage period. However, ethylene significantly inhibited the increase in relative conductivity and MDA content and the decrease in CAT, SOD, and POD activities, thus delaying the occurrence of CI in banana fruits (Fig. 1b).

### Changes in phospholipid levels in control-, cold- and cold ethylene-treated banana fruits

Increasing evidence has demonstrated that polar lipid PA accumulates under low temperatures to form an unstable H_II_-type phase with other lipids, leading to membrane structure shrinkage and ion leakage [71]. This affects the integrity and stability of the cell membrane. To explore the effects of normal temperature (22°C) and cold stress (7°C) with and without ethylene treatment on the levels of phospholipids in banana fruits, we analyzed the profiles of phospholipids in banana fruits at 0, 5, 7 days following treatment using electrospray ionization–tandem mass spectrometry (ESI–MS/MS). As shown in Fig. 1c, compared with the control group, cold stress induced increases in PA and PG content and reduced the contents of PC, PE, and PI. It especially increased the PA content significantly on the 7th day of storage and notably decreased the PI content on the 5th day of storage and PC content on the 5th and 7th days of storage. However, compared with the cold treatment group, exogenous ethylene significantly reduced and increased the PA and PC content, respectively, and improved the contents of PE, PG, and PI. Among all phospholipids, only PG is located in the thylakoid membranes, which are associated with cold stress-induced photosystem (PS) II photoinhibition [72]. Collectively, our findings suggested that, under cold stress, the accumulation of PA correlates well with declines of PC, PE, and PI contents. In addition, ethylene can directly inhibit PA accumulation to enhance the cold tolerance of banana fruits.

### Ethylene inhibits the expression of *MaNAC25*/*28* and phospholipid degradation genes

**Figure 2:**
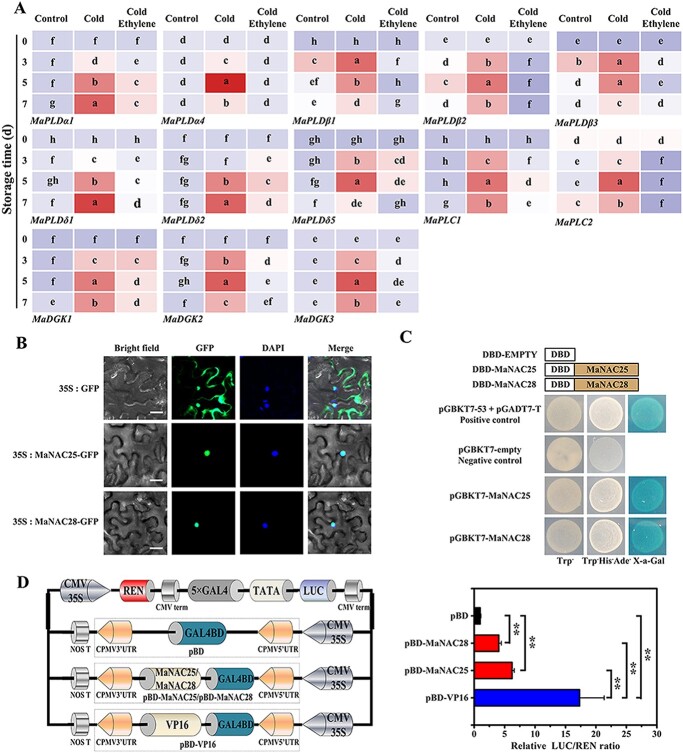
Expression profiles of phospholipid degradation genes and characterization of MaNAC25 and MaNAC28. **a** Expression profiles of eight *MaPLD*s, three *MaDGK*s, and two *MaPLC*s in control banana fruits, cold stress banana fruits, and ethylene treatment banana fruits by RT–qPCR. Total RNA was extracted from pericarp tissues of control fruits, cold stress fruits, and ethylene treatment fruits. Gene transcript levels were normalized against the *MaEIF* gene. Error bars show the standard error from three replicates. Statistical analysis was performed using ANOVA followed by Duncan’s multiple range test at *P* = .05. **b** Subcellular localization of MaNAC25 and MaNAC28 in epidermal cells of tobacco leaves. GFP or MaNAC25-GFP and MaNAC28-GFP were transformed into *N. benthamiana* leaves by *A. tumefaciens* strain EHA105. GFP signal was observed with a fluorescence microscope after 2 days of infiltration. Staining with 4,6-diamidino-2-phenylindole (DAPI) indicates the localization of nuclei. Merged images show colocalization of GFP and DAPI signals. Scale bar = 25 μm. **c** Transcriptional activation of MaNAC25 and MaNAC28 in yeast cells. pGBKT7-MaNAC25 and pGBKT7-MaNAC28 were constructed by inserting the coding region of *MaNAC25* and *MaNAC28* into pGBKT7 (GAL4DBD) and transferred into yeast cells of strain AH109, which were grown on SD plates without tryptophan (Trp−) or without tryptophan, histidine, and adenine (Trp−His−Ade−) for 3 days at 28°C. α-Galactosidase activity was then detected by X-Gal staining. pGBKT7 and pGBKT7–53 + pGADT7-T were used as negative and positive control, respectively. **d** Transactivation of MaNAC25 and MaNAC28 in *N. benthamiana* leaves. The ratio of LUC to REN indicates the transactivation abilities of MaNAC25 and MaNAC28. The ratio of LUC to REN of the empty vector plus promoter vector was used as a calibrator (set to 1). Data are mean ± standard error of six independent biological replicates. ^*^P < .05; ^**^P < .01 compared with pBD (Student’s *t*-test).

PA ensures dynamic homeostasis by participating in plant growth and development and other stress responses [18]. PA is generated via two signaling pathways including the DGK/PLC pathway [15, 18] and the PLD pathway [73]. We used the banana genome (http://banana-genome-hub.southgreen.fr/) [65] and identified 14 *MaPLD*, 9 *MaDGK*, and 13 *MaPLC* genes in bananas. Next, phylogenetic trees of PLDs, DGKs, and PLCs from banana, tomato, and *Arabidopsis* were constructed, respectively. Banana *MaPLD*s, *MaDGK*s, and *MaPLC*s were named according to their evolutionary relationships with their tomato and *Arabidopsis* counterparts. For example, 14 *MaPLD*s were named as *MaPLDα1*/*2*/*3*/*4*, *MaPLDβ1*/*2*/*3*, *MaPLDδ1*/*2*/*3*/*4*/5, and *MaPLDζ1*/*2*, according to their distribution in the α, β/γ, δ, and ζ subfamilies, respectively (Supplementary Data Fig. S1). Nine *MaDGK*s were named *MaDGK1*/*2*/*3*/*4*/*5*/*6*/*7*/*8*/*9*, of which *MaDGK1*/*2*/*6*/*8*/*9* belonged to Cluster I and *MaDGK5* belonged to Cluster II, whereas *MaDGK3*/*4*/*7* belonged to Cluster III (Supplementary Data Fig. S2). Thirteen *MaPLC*s were designated as *MaNPC1*/*2*/*3*/*4*/*5*/*6*/*7* and *MaPLC1*/*2*/*3*/*4*/*5*/*6* based on their clustering in NPC and PI-PLC groups, respectively (Supplementary Data Fig. S3). Heat map analysis (Fig. 1c) and reverse transcription–quantitative polymerase chain reaction (RT–qPCR) screening of changes in phospholipid degradation gene expression showed that the expression of eight *MaPLD* (*MaPLDα1*/*4*, *MaPLDβ1*/*2*/*3*, *MaPLDδ1*/*2*/*5*), three *MaDGK* (*MaDGK1*/*2*/*3*), and two *MaPLC* (*MaPLC1*/*2*) genes were significantly upregulated under cold stress; however, the expression was attenuated by ethylene treatment (Fig. 2a). These results are in good agreement with the PA accumulation data (Fig. 1c).

NAC TFs are involved in responses to several varieties of stress stimulus, such as hypoxia, cold, salt, drought, and plant hormones including ethylene, jasmonic acid, gibberellic acid, ABA, salicylic acid, and indole-3-acetic acid [25–27]. Propylene is known to induce the expression of *MaNAC1*, which consequently modulates the ICE1-CBF cold signaling pathway and enhances the cold tolerance of banana fruit [27]. To investigate whether NAC TFs participated in ethylene-mediated cold tolerance of banana fruits by regulating phospholipid degradation to produce PA, we identified five NAC TFs whose expression patterns were similar to those of phospholipid degradation genes (Supplementary Data Fig. S4). These results indicated that NAC TFs regulated the degradation of phospholipids to produce PA during cold storage. As we successfully obtained *MaMNAC25*-overexpression (OE) /*MaMNAC28*-OE transgenic tomato plants due to the strict screening, MaNAC25 and MaNAC28 were selected for the study. The basic information on MaNAC25 and MaNAC28 is shown in Supplementary Data Table S1. A phylogenetic tree of NAC proteins from banana, *Arabidopsis*, tomato, and grape was created, in which MaNAC25 and MaNAC28 were clustered in subgroups I and VI, respectively (Supplementary Data Fig. S5A). MaNAC25 and MaNAC28 contain a conserved N-terminal NAC domain with five subdomains (A–E) that form a DNA-binding domain and a C-terminal domain responsible for transcription activation, similar to that reported in other NAC TFs [24] (Supplementary Data Fig. S5B). MaNAC25-green fluorescent protein (GFP) and MaNAC28-GFP fusion constructs were transiently expressed in tobacco leaves, which showed their nuclear location (Fig. 2b). As shown in Fig. 2c, yeast cells carrying pGBKT7-MaNAC25, pGBKT7-MaNAC28, and the positive control (pGBKT7-53 + pGADT7-T) exhibited α-galactosidase activity, whereas the negative control (pGBKT7 vector only) did not (Fig. 2c). Furthermore, the values of relative Firefly Luciferase (LUC) /Renin luciferase (REN) of pBD-MaNAC25 and pBD-MaNAC28 were higher than those in the pBD control (Fig. 2d), suggesting that MaNAC25 and MaNAC28 are nuclear proteins with transcription activation capacity.

### MaNAC25 and MaNAC28 positively regulate the transcription of phospholipid degradation genes by binding to their promoters

**Figure 3:**
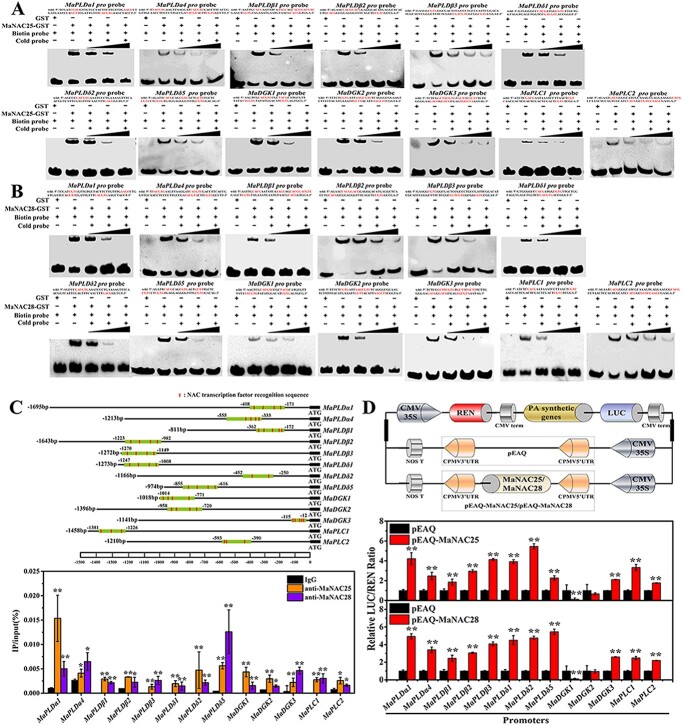
Interaction of MaNAC25/28 with *MaPLDα1*/*4*, *MaPLDβ1*/*2*/*3*, *MaPLDδ1*/*2*/*5*, *MaDGK1*/*2*/*3*, and *MaPLC1*/*2*. **a** Binding of MaNAC25 to *MaPLDα1*/*4*, *MaPLDβ1*/*2*/*3*, *MaPLDδ1*/*2*/*5*, *MaDGK1*/*2*/*3*, and *MaPLC1*/*2* promoters containing the NAC element. **b** Binding of MaNAC28 to *MaPLDα1*/*4*, *MaPLDβ1*/*2*/*3*, *MaPLDδ1*/*2*/*5*, *MaDGK1*/*2*/*3*, and *MaPLC1*/*2* promoters containing the NAC element. GST protein alone was used as a negative control. The sequences of the WT probe containing the NAC element marked with red were biotin-labeled and are shown at the top of the image. ‘−’ represents absence, ‘+’ represents presence. Triangles indicate increasing amounts (100× and 500×) of unlabeled probes for competition experiments. **c** ChIP–qPCR assay showing the binding of MaNAC25/28 to *MaPLDα1*/*4*, *MaPLDβ1*/*2*/*3*, *MaPLDδ1*/*2*/*5*, *MaDGK1*/*2*/*3*, and *MaPLC1*/*2* promoters. DNA samples were immunoprecipitated with MaNAC25/28 antibody or IgG control. The fragments containing NAC binding motifs present in *MaPLDα1*/*4*, *MaPLDβ1*/*2*/*3*, *MaPLDδ1*/*2*/*5*, *MaDGK1*/*2*/*3*, and *MaPLC1*/*2* promoters were marked red and used for analysis. Fold enrichment for antibodies against MaNAC25/28 relative to control IgG is presented as mean ± standard error of three replicates. ^*^P < .05; ^**^P < .01 (Student’s *t*-test). **d** Transient expression assay showing that MaNAC25 and MaNAC28 activate the transcription of *MaPLDα1*/*4*, *MaPLDβ1*/*2*/*3*, *MaPLDδ1*/*2*/*5*, *MaDGK1*/*2*/*3*, and *MaPLC1*/*2*. The LUC/REN ratio of the empty vector (pEAQ) plus promoter was used as calibrator (set as 1). Data are mean ± standard error of six independent biological replicates. ^*^P < .05; ^**^P < .01 (Student’s *t*-test).

It has been reported that NAC TFs can recognize the NAC recognition sequences, containing CA(A/C)G(T/C)(T/C/A)(T/C/A), (T/A)NN(C/T)(T/C/G)TNNNNNNNA(A/C)GN(A/C/T)(A/T), CATGTG, CACG, CGTA, GCTT, and CGTA/G in the promoters of target genes, especially the core sequence CACG [24, 37]. We found that the promoter sequences of 13 phospholipid degradation genes, including *MaPLDα1*/*4*, *MaPLDβ1*/*2*/*3*, *MaPLDδ1*/*2*/*5*, *MaDGK1*/*2*/*3*, and *MaPLC1*/*2*, contained NAC-binding motifs (Supplementary Data Text S1). Electrophoretic mobility shift assays (EMSAs) were conducted to detect the binding of MaNAC25 and MaNAC28 with these promoters. The results showed that the recombinant MaNAC25-Glutathione-S-transferase (GST) and MaNAC28-GST proteins (Supplementary Data Fig. S6), and not the GST protein, efficiently bound to the promoters of these 13 phospholipid degradation genes (Supplementary Data Text S1; Fig. 3a and b). These binding bands gradually weakened when cold probes were added at increasing concentrations (Fig. 3a and b). Moreover, we confirmed the binding of MaNAC25 and MaNAC28 to these promoters by chromatin immunoprecipitation (ChIP)–qPCR analysis using anti-MaNAC25 and anti-MaNAC28 antibodies. Compared with the negative control IgG, the regions containing NAC-binding motifs in these promoters were significantly enriched by anti-MaNAC25 and anti-MaNAC28 antibodies (Fig. 3c), which is consistent with the EMSA results. To further assess the ability of MaNAC25 and MaNAC28 to regulate the transcription of 13 phospholipid degradation genes, we conducted transient expression assays in tobacco leaves. Of the 13 promoters tested, MaNAC25 and MaNAC28 significantly activated those of *MaPLDα1*/*4*, *MaPLDβ1*/*2*/*3*, *MaPLDδ1*/*2*/*5*, and *MaPLC1*/*2* genes but not those of *MaDGK1*/*2*, as evident from the LUC/REN ratio (Fig. 3d).

**Figure 4:**
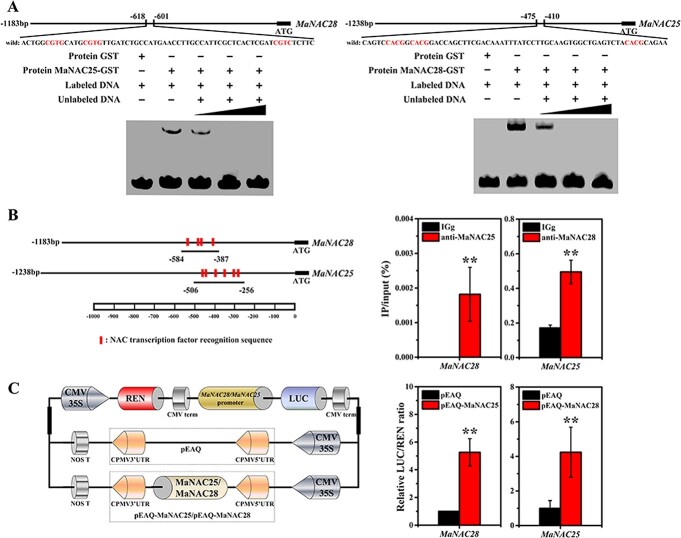
MaNAC25 and MaNAC28 perform positive feedback regulation. **a** EMSA showing that both MaNAC25 and MaNAC28 effectively bound to each other’s promoters. GST protein alone was used as a negative control. The sequences of the WT probe containing the NAC element marked with red were biotin-labeled and shown at the top of the image. ‘−’ represents absence, ‘+’ represents presence. Triangles indicate increasing amounts (100× and 500×) of unlabeled probes for competition experiments. **b** ChIP–qPCR assay showing that both MaNAC25 and MaNAC28 effectively bound to each other’s promoters. DNA samples were immunoprecipitated with MaNAC25 and MaNAC28 antibody or IgG control. Fragments containing NAC binding motifs present in *MaNAC28* and *MaNAC25* promoters were marked red and used for analysis. Fold enrichment for antibodies against MaNAC25 and MaNAC28 relative to control IgG is presented as mean ± standard error of three replicates. ^*^P < .05; ^**^P < .01 (Student’s *t*-test). **c** Transient expression assay showing that MaNAC25 activated *MaNAC28* transcription and MaNAC28 activated *MaNAC25* transcription. The LUC/REN ratio of the empty vector (pEAQ) plus promoter was used as calibrator (set as 1). Data are mean ± standard error of six independent biological replicates. ^*^P < .05; ^**^P < .01 (Student’s *t*-test).

Increasing evidence has showed that TFs can form a feedback loop to modulate plant growth and development under several stress stimuli [74, 75]. Thus, we speculated that MaNAC25 and MaNAC28 might form a feedback loop as well. We performed EMSA, ChIP–qPCR, and transient expression analyses to validate this. The promoter sequences of *MaNAC25* and *MaNAC28* contain the NAC core element CACG (Supplementary Data Text S1). EMSA and ChIP–qPCR showed that both MaNAC25 and MaNAC28 effectively bound to each other’s promoters (Fig. 4a and b). Transient expression analysis further demonstrated that both MaNAC25 and MaNAC28 significantly activated each other’s promoters (Fig. 4c). To summarize, MaNAC25 and MaNAC28 form a positive feedback loop to induce the transcription of phospholipid degradation genes and PA production.

### Overexpression of *MaNAC25* and *MaNAC28* enhances cold sensitivity of tomato fruits, which can be offset by ethylene treatment

**Figure 5:**
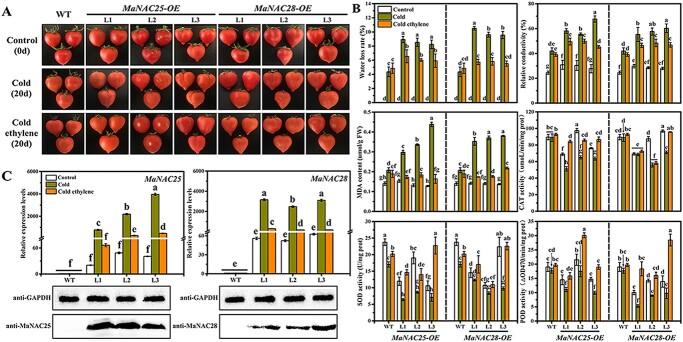
Ethylene reduces the cold sensitivity of tomato fruits overexpressing *MaNAC25* and *MaNAC28***. a** Phenotypes of tomato fruits overexpressing *MaNAC25* and *MaNAC28* in control, cold, and cold ethylene treatment. Tomato fruits from WT and transgenic lines at color-breaking stage were sampled as control fruits (Control, 0 d). Fruits from the same period were stored at 4°C for 20 days and sampled as cold-treated fruits (Cold, 20 d). Fruits from the same period treated with ethylene (0.04% ethephon) were stored at 4°C for 20 days and sampled as cold ethylene-treated fruits (Cold ethylene, 20 d). **b** Changes in water loss rate, relative conductivity, MDA content and CAT, SOD, and POD activity assays in WT and transgenic tomato fruits. Error bars show the standard error from three replicates. Statistical analysis was performed using ANOVA followed by Duncan’s multiple range test at *P* = .05. **c** Expression of *MaNAC25* and *MaNAC28* in WT and transgenic tomato fruits by RT–qPCR. Total RNA was extracted from pericarp tissues of WT and transgenic tomato fruits. The gene transcript levels were normalized against the *Actin* gene, followed by normalization against WT expression. Error bars show the standard error from three replicates. Statistical analysis was performed using ANOVA followed by Duncan’s multiple range test at *P* = .05. Western blotting experiment confirmed that *MaNAC25* and *MaNAC28* were successfully transferred into the tomato. Total protein was extracted from pericarp tissues of WT and transgenic lines. Anti-GAPDH antibody was used as a reference control to normalize the loading proteins.

Recently, increasing evidence has demonstrated the involvement of NAC TFs in several stress stimuli. In addition, the overexpression of NAC TFs, including maize *ZmNAC33*, rice *ONAC106* and *ONAC022*, tomato *SlJUB1*, *SlNAC4*, *SlNAC1*, and *SlNAM1*, improved the tolerance of transgenic plants to dehydration, cold, drought, and salt [28, 30, 33, 35, 36, 44, 45]. To explore the function of MaNAC25 and MaNAC28 in relation to CI in banana fruit, *MaNAC25* and *MaNAC28* were overexpressed in Micro-Tom tomato fruits. Immunoblotting experiments confirmed that the *MaNAC25* and *MaNAC28* genes were successfully transferred and expressed in tomato fruits (Fig. 5c). We found that cold stress caused an earlier appearance of CI in tomato fruits overexpressing either *MaNAC25* or *MaNAC28* than wild-type (WT) fruits, with CI occurring at 20 and 33 days after storage in transgenic and WT fruits, respectively (Supplementary Data Fig. S7; Fig. 5a). This finding indicated that overexpression of *MaNAC25* or *MaNAC28* increased the sensitivity of tomato fruits to cold stress. Ethylene can alleviate CI in banana [27, 52] and tomato fruits [49]. In this work, tomato fruits overexpressing *MaNAC25* and *MaNAC28* were pretreated with ethylene (0.04% ethephon) and then stored under cold stress (4°C) for ~40 days. The transgenic tomato fruits underwent color darkening after 20 days of storage at 4°C, and the CI symptoms became severe with extended storage. By contrast, transgenic tomato fruits pretreated with ethylene developed CI at 33 days of storage, whereas ethylene-treated WT fruits maintained a healthy appearance with only mild CI symptoms after 40 days of storage (Supplementary Data Fig. S7). These results demonstrated that ethylene reduced the cold sensitivity of tomato fruits conferred by the overexpression of *MaNAC25* and *MaNAC28*.

In addition, compared with control fruits, cold stress significantly increased the water loss rate, relative conductivity, and MDA content and weakened CAT, SOD, and POD activities in WT and transgenic tomato fruits, and overexpressing tomato fruits had lower levels of water loss rate, relative conductivity, and MDA content and higher levels of CAT, SOD, and POD activities than WT fruits under cold stress. However, ethylene treatment alleviated CI in tomato fruits overexpressing *MaNAC25* and *MaNAC28*, with lower water loss rate, relative conductivity, and MDA content, and higher CAT, SOD, and POD activities (Fig. 5b). Furthermore, cold stress significantly increased the expression of *MaNAC25* and *MaNAC28*, whereas ethylene treatment inhibited it (Fig. 5c). These findings were similar to the results found in bananas.

### Ethylene inhibits cold-induced upregulation of phospholipid degradation genes and PA accumulation in tomato fruits overexpressing *MaNAC25* and *MaNAC28*

**Figure 6:**
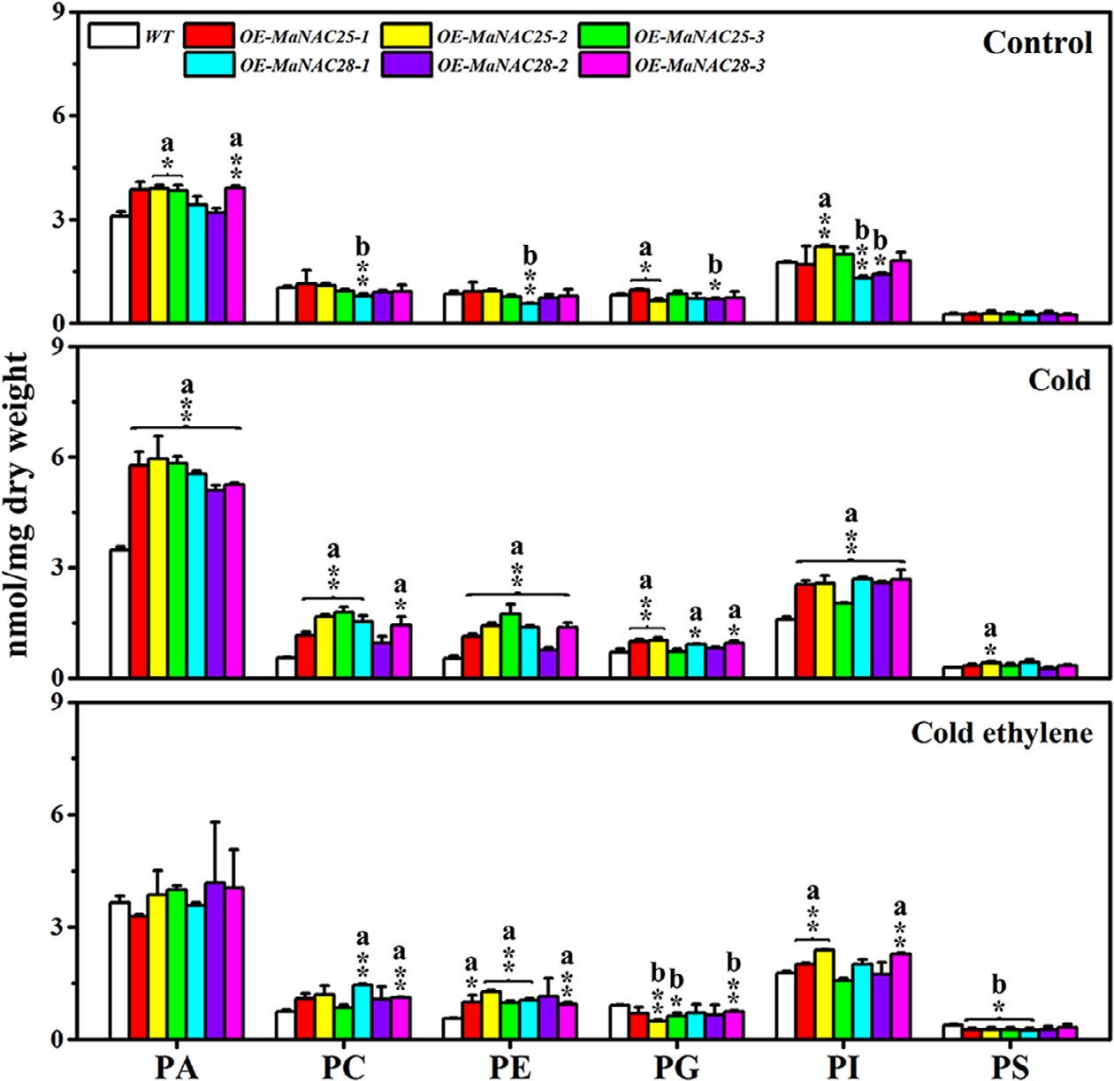
Total lipid content in WT and transgenic tomato fruits. Values are represented as mean ± standard error (nmol/mg dry weight; *n* = 4). Asterisks indicate significant differences from WT fruit (^*^P < .05; ^**^P < .01, Student’s *t*-test). ^a^Values were higher when compared with WT fruit in the same experiment (^*^P < .05; ^**^P < .01). ^b^Values were lower compared with WT fruit in the same experiment (^*^P < .05; ^**^P < .01).

We next measured the phospholipid components in WT and transgenic tomato fruits under cold stress. As shown in Fig. 6, the content of PA increased slightly and that of PC, PE, PI, and PG decreased slightly in WT fruits under cold stress, whereas ethylene treatment reversed cold-induced changes in phospholipid content. However, the levels of PA, PC, PE, PG, and PI in *MaNAC25*-OE and *MaNAC28*-OE lines were significantly higher than those in control transgenic lines or WT fruits under cold stress (Fig. 6). Further, PE and PI levels in *MaNAC25*-OE and PC, PE, and PI levels in *MaNAC28*-OE lines were significantly increased in comparison with those in WT fruits and in ethylene-treated fruits; however, these levels were lower than those in transgenic lines under cold stress. These data suggest that the overexpression of *MaNAC25* and *MaNAC28* in tomatoes significantly altered the content of phospholipids under cold stress. This could be attributed to the excessive accumulation of PA, which accelerates CI in transgenic tomato fruits, whereas ethylene treatment inhibits cold-induced PA accumulation, consequently delaying the occurrence of CI in transgenic tomato fruits.

**Figure 7:**
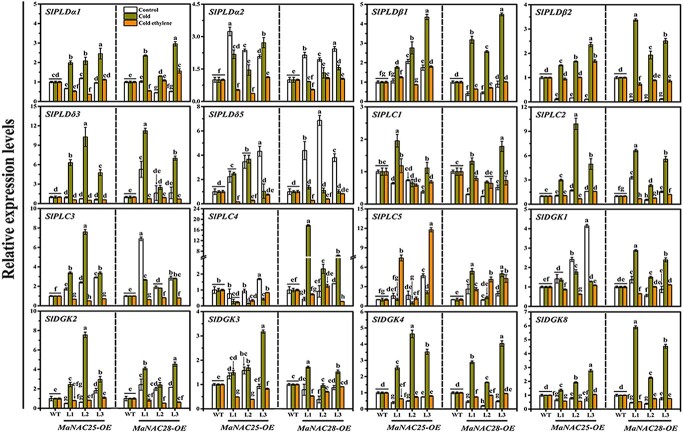
Relative expression levels of phospholipid degradation genes (*SlPLDα1*/*4*, *SlPLDβ1*/*2*, *SlPLDδ3*/*5*, *SlPLC1*/*2*/*3*/*4*/*5*, and *SlDGK1*/*2*/*3*/*4*/*8*) in WT and transgenic tomato fruits. Gene expression levels were determined by RT–qPCR. Gene transcript levels were normalized against the *Actin* gene, followed by normalization against WT expression. Error bars show the standard error from three replicates. Statistical analysis was performed using ANOVA followed by Duncan’s multiple range test at *P* = .05.

We next analyzed the expression levels of phospholipid degradation genes in WT and transgenic lines. A total of 16 genes responsible for phospholipid degradation to produce PA in tomatoes, including *SlPLDα1*/*4*, *SlPLDβ1*/*2*, *SlPLDδ3*/*5*, *SlPLC1*/*2*/*3*/*4*/*5*, and S*lDGK1*/*2*/*3*/*4*/*8*, were selected (Supplementary Data Table S2). Compared with the control fruits, the expression of *SlPLDα1*, *SlPLDβ1*/*2*, *SlPLDδ3*, *SlPLC1*/*2*/*3*, and *SlDGK2*/*3*/*4*/*8* in *MaNAC25*-OEs, as well as that of *SlPLDα1*, *SlPLDβ1*/*2*, *SlPLDδ3*, *SlPLC1*/*2*/*4*/*5*, and *SlDGK1*/*2*/*3*/*4*/*8* in *MaNAC28*-OEs, was significantly upregulated under cold stress (Fig. 7). In addition, expression was considerably higher than that in WT fruits. As expected, exogenous ethylene treatment significantly inhibited the cold-induced upregulation of these genes, similar to the expression pattern of phospholipid degradation genes in banana fruits under the same conditions.

## Discussion

Increasing evidence indicates that cold stress-induced excessive PA accumulation can result in the formation of unstable hexagonal II (H_II_) phases [23, 71], which disrupt the integrity and stability of cell membranes [1, 23]. We found that cold stress significantly induced PA accumulation, accompanied by a decrease in PC, PE, and PI (Fig. 1c). We believe that cold stress promoted the hydrolysis of PC, PE, and PI to generate high levels of PA, which induced CI in banana fruits. However, ethylene treatment inhibited the changes in phospholipid levels induced by cold stress, especially PA accumulation, thus retaining the stability of cell membranes and preventing damage to banana fruits. Our findings illustrated ethylene-mediated inhibition of PA accumulation as an important mechanism to enhance cold tolerance in banana fruits. In addition, the 13 genes responsible for phospholipid degradation to produce PA in bananas were significantly upregulated and inhibited by cold stress and ethylene treatment, respectively, which was consistent with the changes in PA levels (Fig. 1c), indicating that these genes function in lipid metabolism during cold stress.

The latest research reported that CaNAC1 promotes PA accumulation in green bell pepper by regulating the degradation of phospholipids at low temperatures [48]. However, the regulatory mechanism remains unclear. Moreover, the regulation of phospholipid degradation to produce PA at the transcriptional level has been rarely reported in fruits and model plants such as tomato and *Arabidopsis* fruits. In this study, we demonstrated that ethylene-mediated increased tolerance to cold stress in banana fruits was intricately related to ethylene-inhibited phospholipid degradation to produce PA. Furthermore, we identified two nuclear NAC TFs, namely, MaNAC25 and MaNAC28, that belong to two different subgroups (Fig. 2b; Supplementary Data Fig. S5A). Although they were highly expressed following cold stress (Supplementary Data Fig. S4), ethylene abolished their induction, a finding parallel with the expression pattern of phospholipid degradation genes. The above results suggested that MaNAC25 and MaNAC28 are involved in phospholipid degradation to produce PA, at least in part, by modulating the expression of phospholipid degradation genes. Furthermore, MaNAC25 and MaNAC28 can directly bind to the promoters of 13 phospholipid degradation genes both *in vivo* and *in vitro* (Fig. 3a–c). MaNAC25 and MaNAC28 were shown to activate the expression of *MaPLDα1*/*4*, *MaPLDβ1*/*2*/*3*, *MaPLDδ1*/*2*/*5*, *MaDGK3*, and *MaPLC1*/*2* (Fig. 3d). Interestingly, MaNAC25 and MaNAC28 did not affect the transcription of *MaDGK1*/*2*, implying that *MaDGK1*/*2* is regulated by other transcriptional regulators. Our findings suggested that MaNAC25 and MaNAC28 promoted PA accumulation via the PLD pathway by activating the expression of *MaPLDα1*/*4*, *MaPLDβ1*/*2*/*3*, and *MaPLDδ1*/*2*/*5*, especially *MaPLDα1* and *MaPLDδ5*, or through the DGK/PLC pathway by activating the expression of *MaDGK3* and *MaPLC1*/*2*. Furthermore, ethylene inhibited cold-induced upregulated expression of *MaNAC25* and *MaNAC28*, as well as that of phospholipid degradation genes. Thus, our results illustrated that ethylene not only directly inhibited the degradation of phospholipids, thus preventing PA accumulation, but also indirectly reduced the MaNAC25 and MaNAC28-mediated activation of phospholipid degradation genes.

NAC TFs regulate plant responses to several environmental stresses. Several NAC TFs, such as tomato SlNAC1 and SlNAM1, are known to positively regulate cold tolerance [44, 45]. However, NACs can also negatively regulate cold tolerance, e.g. ShNAC1 from the wild tomato species *S. habrochaites* [47]. Although CaNAC1 from green pepper regulates phospholipid degradation to produce PA during cold stress [48], it is unknown whether NAC TFs can negatively regulate fruit cold tolerance by modulating phospholipid degradation to produce PA. In this research, we revealed that MaNAC25 and MaNAC28 negatively regulate cold tolerance in banana fruits by positively modulating phospholipid degradation to produce PA. In addition, the overexpression of *MaNAC25* and *MaNAC28* in tomatoes enhanced the cold sensitivity of tomato fruits (Fig. 5a), as indicated by the higher levels of water loss rate, relative conductivity, MDA content, and accumulation of PA, PE, PC, and PI (Figs 5b and 6), as well as lower levels of CAT, SOD, and POD activities (Fig. 5b). These findings could be ascribed to cold stress-induced transcriptional activation of MaNAC25 and MaNAC28, which, in turn, activated phospholipid degradation genes and promoted PA accumulation and CI development in transgenic tomato fruits (Figs 5c and 7). However, we believe that the increased PE, PC, and PI contents induced by cold stress in transgenic tomato fruits can continuously supply the substrates for phospholipid degradation to produce PA. Interestingly, ethylene treatment repressed enhanced cold sensitivity in transgenic tomatoes overexpressing *MaNAC25* and *MaNAC28* (Fig. 5a). In particular, the levels of PA, PC, PE, PI, PG, and PS in transgenic tomato fruits reduced following treatment with ethylene (Fig. 6). Strikingly, ethylene treatment inhibited the cold stress-induced accumulation of PA in transgenic tomatoes. However, the levels of PE and PI in *MaNAC25*-OE lines as well as those of PC, PE, and PI in *MaNAC28*-OE lines were significantly higher than those in WT fruits. We speculated that ethylene primarily attenuated the transcriptional activation of PLD genes by MaNAC25 and MaNAC28 through the PLD pathway and *DGK*/*PLC* genes in transgenic tomato fruits to inhibit PA accumulation and maintain higher levels of PE, PC, and PI (the substrates of PA production).

TFs are known to form a feedback loop during plant growth and development and in response to several stress stimuli. For instance, MaMYB3 inhibits the expression of *MabHLH6* to reduce the activation of starch degradation by MabHLH6, thus delaying banana fruit ripening [74]. Similarly, in tomato fruits, SlAN2-like, an R2R3-MYB TF, activates the expression of *SlMYBATV* to balance the anthocyanin content [75]. In *Arabidopsis*, NAC016 and NAC-like, activated by AP3/PI (NAP), comodulate the downstream genes in response to drought stress [42]. Furthermore, recent research indicated that banana vascular-related NAC domain (VND)1/2/3 regulate the transcription of *MusaVND6* and *MusaVND7* by binding to a 19-bp secondary-wall NAC-binding element (SNBE)-like motif [37]. We demonstrated that MaNAC25 and MaNAC28 formed a positive feed-forward loop to regulate the transcription of each other. In this feedback loop, MaNAC25 activated the transcription of *MaNAC28* to enhance the transcriptional activation of phospholipid degradation genes by MaNAC28. Moreover, MaNAC28 activated the transcription of *MaNAC25* to promote the transcriptional activation of MaNAC25, forming a complete regulatory loop in PA production during banana CI (Fig. 4). Based on these results, it would be interesting to explore whether MaNAC25 and MaNAC28 can form a feed-forward loop with other NAC TFs or other TFs to co-regulate phospholipid degradation to produce PA. In addition, their ability to form homo- and heterodimers with other proteins to participate in phospholipid degradation to produce PA can be explored. To our knowledge, this is the first study to report that NAC TFs co-regulate ethylene-mediated cold tolerance by modulating phospholipid degradation to produce PA in banana fruits.

**Figure 8:**
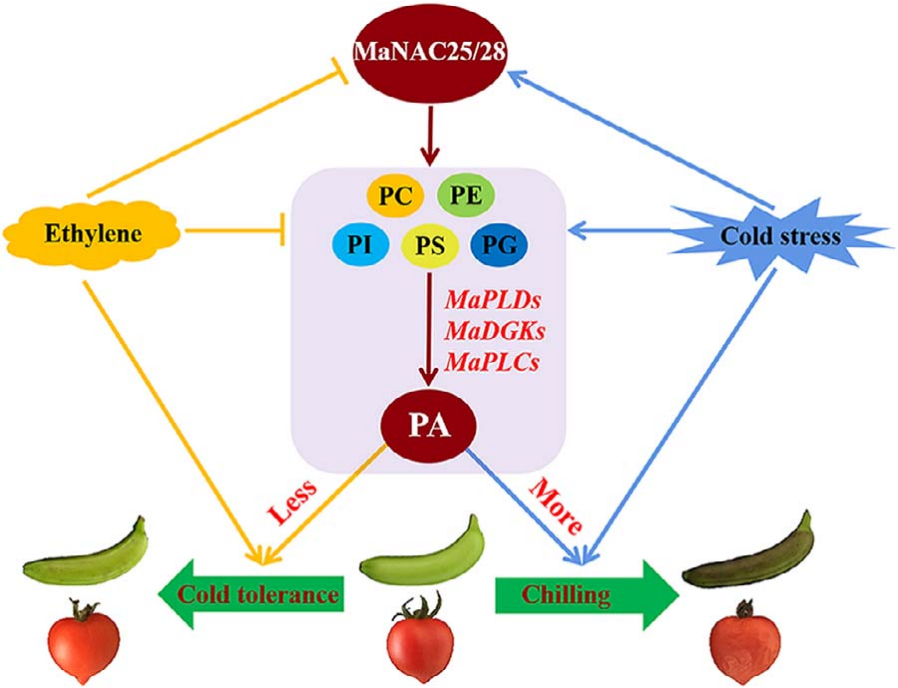
A working model that states that NAC TFs regulate ethylene-mediated cold tolerance by modulating the degradation of phospholipids in banana and transgenic tomato fruits. Cold stress significantly induced the upregulated expression of *MaNAC25* and *MaNAC28* and phospholipid degradation genes (*PLD*s, *DGK*s, and *PLC*s), resulting in phospholipid degradation to produce PA and CI in banana and transgenic tomato fruits. However, ethylene not only directly inhibited the degradation of phospholipids in banana and transgenic tomato fruits, but also reduced the activity of MaNAC25 and MaNAC28, thereby inhibiting the degradation of phospholipids. Meanwhile, MaNAC25 and MaNAC28 formed a positive feedback loop to activate the transcription of each other. The arrow indicates activation, while the T bar represents repression.

In conclusion, we proposed a hypothetical working model that states that MaNAC25 and MaNAC28 regulate ethylene-mediated cold tolerance by modulating phospholipid degradation to produce PA in banana and transgenic tomato fruits (Fig. 8). During cold stress, MaNAC25 and MaNAC28 directly or indirectly activate the transcription of phospholipid degradation genes to induce PA production and CI development in banana fruit. These results were confirmed in transgenic tomato fruits overexpressing *MaNAC25* and *MaNAC28*. In addition, ethylene directly inhibits phospholipid degradation to produce PA, and indirectly reduces phospholipid degradation to produce PA by inhibiting the activation of phospholipid degradation genes by MaNAC25 and MaNAC28 in banana and transgenic tomato fruits via a positive feedback loop. We believe this research will strengthen our understanding of the co-regulatory mechanism of NACs in phospholipid degradation to produce PA, and membrane lipid remodeling to negatively regulate cold tolerance in certain cold-sensitive fruits.

## Materials and methods

### Plant materials and treatments

Banana (*Musa acuminata* AAA, cv. ‘Cavendish’) fruits were harvested at commercial maturity from a local orchard in Guangzhou, southeastern China. The fruits were divided into individual fingers, and then selected for uniform maturity, shape, and weight, and absence of any damage. The selected fruits were distributed into three treatment groups: two groups were immersed in sterile deionized water for 1 minute, and then stored at 22°C and 7°C, respectively, as the control group and the cold treatment group. The third group was dipped in 500 mg/l ethephon aqueous solution for 1 minute and stored at 7°C, as the cold ethylene treatment group. All treated fruits were packed in bags after being air-dried, and stored at the corresponding temperature with 90% relative humidity for 7 days. Banana peels were taken at 0, 3, 5 and 7 days after storage for the determination of relevant indicators. All samples were frozen in liquid nitrogen and placed at −80°C for standby.

### Determination of relative conductivity and MDA content

The relative conductivity, which reflects cell membrane permeability, was measured using the previously described method [59]. Membrane oxidation was evaluated by the determination of MDA content as previously described [60].

### Enzyme activity assay

CAT (G0105W), SOD (G0101W), and POD (G0107W) activities were measured using the enzyme activity assay kit of Suzhou Grace Biotechnology Co., Ltd. (www.geruisi-bio.com) according to the manufacturer’s instructions.

### Lipid profiling

Total lipid extraction, profiling, and normalization were conducted as reported previously [23, 61] with minor modification. Briefly, samples of the same size, including banana peel and the conjoined parts of tomato peel and pulp, were transferred to 4 ml of isopropanol with 0.01% butylated hydroxytoluene at 75°C for 15 minutes. After cooling, the samples were placed at −80°C until all of the samples were ready for lipid extraction. After thawing in the dark, 12 ml of chloroform:methanol:300 mM ammonium acetate (30:41.5:3.5) was added and the sample was shaken slowly for 24 hours (100 × g). After standing for 20 minutes, all extracts were transferred to 10 ml glass tubes and then evaporated under nitrogen after removing the water phase. Finally, the lipid extract was dissolved in 1 ml of chloroform for the assay. The remaining sample tissue was dried overnight at 105°C and weighed, giving dry weights for subsequent calculations. The standardization and determination of total lipid content were conducted by ESI–MS/MS as reported previously [23, 62].

### RNA preparation, RT–PCR, and RT–qPCR

RNA preparation, RT–PCR and RT–qPCR reactions were conducted using the same methods as previously reported [63].

### Subcellular location assay

MaNAC25-GFP and MaNAC28-GFP fusion plasmids were generated by subcloning the coding sequences (CDSs) of *MaNAC25*/*28* without the terminator codon into the pEAQ-HT-GFP vector for subcellular localization assay. The transient expression of MaNAC25-GFP, MaNAC28-GFP, and control GFP were conducted in tobacco (*N. benthamiana*) leaves via *Agrobacterium*-mediated infiltration as previously reported [64]. The infiltrated tobacco plants were cultured at 22°C for 48 hours for further analysis. GFP fluorescence signal was visualized and photographed with a fluorescence microscope.

### Transcriptional activation assay in yeast cells

The pGBKT7-MaNAC25 and pGBKT7-MaNAC28 vectors were generated by cloning the CDS of *MaNAC25*/*28* into the pGBKT7 vector (Clontech). These vectors were transformed into yeast strain AH109 together with positive and negative controls, respectively, and then streaked on plates containing SD medium without tryptophan (SD/−Trp) and without tryptophan, histidine, and adenine (SD/−Trp−His−Ade) at 30°C for 3 days. The transcriptional activation of MaNAC25/28 was evaluated based on the growth situation and α-galactosidase activity following the manufacturer’s guidelines.

### Cloning and analysis of promoters

Based on sequences in the banana genome-wide sequence database [65], the promoters of *MaNAC25*, *MaNAC28*, and phospholipid degradation genes were isolated and analyzed.

### Dual-luciferase transient expression assay

pBD-MaNAC25 and pBD-MaNAC28 were generated by cloning the CDS of *MaNAC25*/*28* into pBD vector for a transcriptional activity assay [66]. To examine the binding activities of MaNAC25/28 on the promoters of phospholipid degradation genes, pEAQ-MaNAC25 and pEAQ-MaNAC28 were constructed by cloning the full lengths of *MaNAC25*/*28* into pEAQ as effectors, while the promoters of phospholipid degradation genes were cloned into the pGreenII 0800-LUC vector as reporter [66]. Transient expression of the constructed plasmids was conducted in tobacco leaves. The activities of LUC and REN were analyzed, and the LUC/REN ratio was calculated to display the transcriptional activation activities of MaNAC25/28 on the transcription of phospholipid degradation genes.

### Protein expression and electrophoretic mobility shift assay

The purification of GST-MaNAC25 and GST-MaNAC28 was performed as previously described [67]. The CDSs of *MaNAC25* and *MaNAC28* were cloned into pGEX-4 T-GST. The constructed plasmids of GST-MaNAC25 and GST-MaNAC28, along with GST alone, were individually transformed into *Escherichia coli* strain BM Rosetta (DE3) cells. The recombinant proteins were expressed after induction with isopropyl-β-d-thiogalactopyranoside (IPTG) at 37°C, and then purified with glutathione beads (Clontech). Finally, these proteins were judged for size and purity by SDS–PAGE and Coomassie Brilliant Blue staining.

Probes, including the NAC-binding sites that originated from the promoters of *MaNAC25*, *MaNAC28*, and PA-producing genes, were synthesized and labeled with biotin using a DNA 3′ End Biotinylation Kit (Thermo Fisher Scientific). The unlabeled probes were prepared by annealing complementary oligonucleotides. EMSA was performed by using a LightShift Chemiluminescent EMSA Kit (Thermo Fisher Scientific).

### Chromatin immunoprecipitation–qPCR analysis

Banana peels were cross-linked with 1% formaldehyde for 10 minutes under vacuum, and the banana chromatin complex was extracted and sonicated as previously described [68]. Anti-MaNAC25 and anti-MaNAC28 polyclonal antibodies and Protein A Agarose/Salmon Sperm DNA (Millipore) were used for immunoprecipitation. The immunoprecipitated DNA was analyzed by RT–qPCR after reverse cross-linking, protein digestion, and purification. Anti-MaNAC25 and anti-MaNAC28 antibodies were prepared by Hua An Biotechnology Company.

### Tomato genetic transformation

The CDSs of *MaNAC25* and *MaNAC28* were cloned into the plant binary vector pLP100-35S with a β-glucuronidase (GUS) reporter gene to generate overexpression vectors LB-35S:GUS-35S:MaNAC25-RB and LB-35S:GUS-35S:MaNAC28-RB. The constructs were transformed into Micro-Tom tomato fruits by *Agrobacterium*-mediated transformation following the previously described method [69, 70]. The positive transformants were selected by resistance screening and confirmed by detecting *MaNAC25* and *MaNAC28* expression by RT–qPCR and their proteins by western blotting. Three independently transformed lines (T2) with *MaNAC25* and *MaNAC28* overexpression were used for experiments. Tomato fruits from WT and transgenic lines at color-breaking stage were harvested as control fruits. The fruits in the same period were further divided into two groups: one group was stored at 4°C for 20 days, as cold-treated fruits, and the other group was stored at 4°C for 20 days after ethylene (0.04% ethephon) treatment, as ethylene-treated fruits. Three sets of samples were used for subsequent experimental analysis.

### Water loss rate measurements

Five tomato fruits from the WT and transgenic tomato fruits were detached at color-breaking stage and stored at 4°C for 20 days. Water loss rate was calculated after measuring the weight decrease over time.

### Statistical analysis

The study was designed according to the random principle. Variance analyses were performed using SPSS software, using Duncan’s multiple range test at the *P* < .05 or *P* < .01 level. The means ± standard errors from at least three replicates are presented.

### Primers

The primers used in this research are listed in Supplementary Data Table S3.

## Acknowledgements

We thank Dr George P. Lomonossoff (Department of Biological Chemistry, John Innes Centre, Norwich Research Park, UK) for providing the pEAQ vectors. We also thank Dr Wei Deng (School of Life Sciences, Chongqing University) for assisting in the functional verification of transgenic tomatoes. We also thank Dr Shi Xiao and Dr Qinfang Chen (School of Life Science, Sun Yat-sen University) for lipid determination. This work was supported by the National Natural Science Foundation of China (32102449).

## Author contributions

Z.Y. and C.S. conceived and supervised the project; Z.Y. and C.S. planned and designed the research; C.S. performed most of the experiments and analyzed the data; M.W., Y.Z., Z.G., W.Y., and Y.Z. carried out some of the experiments; C.S. wrote the manuscript; Z.Y. gave advice and edited the manuscript.

## Data availability

The data used to support the findings of this study are available from the corresponding author upon request. The sequence data discussed in this article can be found in the banana Genome Initiative database (http://banana-genome-hub.southgreen.fr/) and tomato Genome Initiative database (Solanum lycopersicum ITAG4.0) under the following accession numbers: *MaNAC25* (Ma06_g33960), *MaNAC28* (Ma09_g09630), *MaPLDα1* (Ma10_g26900), *MaPLDα4* (Ma10_g23910), *MaPLDβ1* (Ma05_g22560), *MaPLDβ2* (Ma04_g31450), *MaPLDβ3* (Ma08_g11770), *MaPLDδ1* (Ma07_g24920), *MaPLDδ2* (Ma11_g01110), *MaPLDδ5* (Ma07_g18790), *MaDGK1* (Ma02_g22740), *MaDGK2* (Ma03_g02590), *MaDGK3*(Ma06_g05490), *MaPLC1* (Ma02_g20790), *MaPLC2* (Ma01_g22520), *SlDGK1* (Solyc12g039180), *SlDGK2* (Solyc05g056420), *SlDGK3* (Solyc07g006580), *SlDGK4* (Solyc06g009270), *SlDGK8* (Solyc03g115370), *SlPLDα1* (Solyc06g068090), *SlPLDα2* (Solyc03g116620), *SlPLDβ1* (Solyc08g080130), *SlPLDβ2* (Solyc01g091910), *SlPLDδ3* (Solyc04g082000), *SlPLDδ5* (Solyc02g061850), *SlPLC1* (Solyc10g076710), *SlPLC2* (Solyc06g051620), *SlPLC3* (Solyc05g052760), *SlPLC4* (Solyc06g007120), *SlPLC5* (Solyc06g051630).

## Conflict of interest

The authors declare no competing interests.

## Supplementary data

Supplementary data is available at *Horticulture Research* online.

## Supplementary Material

Web_Material_uhac039
