# Supplementary material for: NAC-mediated membrane lipid remodeling negatively regulates fruit cold tolerance
Source: Hortic Res. 2022 May 4;9:uhac039. doi: 10.1093/hr/uhac039 (PMC9071380; doi:10.1093/hr/uhac039)
Supplement: Web_Material_uhac039 [file web_material_uhac039.zip › Supporting Data.doc]

**Supporting Data**

**Fig. S1 Phylogenetic relationships of PLDs proteins from banana, tomato, and Arabidopsis.** The phylogenetic tree was drawn using the neighbor-joining method with 1000 bootstrap replicates by MEGA 6.0.

**Fig. S2 Phylogenetic relationships of DGKs proteins from banana, tomato, and Arabidopsis.** The phylogenetic tree was drawn using the neighbor-joining method with 1000 bootstrap replicates by MEGA 6.0.

**Fig. S3 Phylogenetic relationships of PLCs proteins from banana, tomato, and Arabidopsis.** The phylogenetic tree was drawn using the neighbor-joining method with 1000 bootstrap replicates by MEGA 6.0.

**Fig. S4 Changes in gene expression of 5 NAC TFs (*MaNAC28/18/21/23/25*) originally selected in banana fruit by qRT-PCR.** Total RNA was extracted from pericarp tissues of banana fruit. The gene transcript levels were normalized against the *MaEIF* gene. Error bars show SE from three replicates. Statistical analysis was performed using the method of ANOVA, followed by Duncan’s multiple range test at *p* = 0.05.

**Fig. S5 Phylogenetic and sequence analyses of MaNAC25 and MaNAC28.** **A** Phylogenetic relationships of MaNAC25 and MaNAC28 with the NAC proteins from tomato (SlNACs), Arabidopsis (AtNACs) and grape (VvNACs). The phylogenetic tree was drawn using the neighbor-joining method with 1000 bootstrap replicates by MEGA 6.0. **B** Multiple sequence alignment of the conserved NAC domain of MaNAC25 and MaNAC28 with NACs proteins in tomato fruit from the same subgroup.

**Fig. S6 The purification of the recombinant MaNAC25-GST protein (A) and MaNAC28-GST protein (B).**

**Fig. S7 Cold-sensitive phenotypes of tomato fruits overexpressing *MaNAC25* and *MaNAC28* and their control by ethylene.** Tomato fruits from wild-type (WT) and transgenic lines at color-breaking stage were sampled as control group. The cold stress group and ethylene treatment group were stored at 4 ℃ for 40 days, and the photographs were captured and the phenotypic was observed on days 20, 26, 33, and 40, respectively.

**Table S1. Summary of primers used in this study.**

| Primer name | Primer sequence | use |
| --- | --- | --- |
| *MaEIF5A -2-F* | *ACACCAAAGATGATCTGAGGCT* | Quantitative real-time PCR analysis |
| *MaEIF5A -2-R* | *AAGGAACACCAGGGCAACAGT* |  |
| *MaPLDα1-F* | *TCATGGCGAGAGCTATACTCAA* |  |
| *MaPLDα1-R* | *GGATCTTAGCATCTCCAACAGG* |  |
| *MaPLDα4-F* | *CTACGTTCACTCCAAGCTGATG* |  |
| *MaPLDα4-R* | *CTAAAGCCATGGACTTCTCCAC* |  |
| *MaPLDβ1-F* | *GTGGAGCATGTGACTTCGATAA* |  |
| *MaPLDβ1-R* | *GGATCGAAACAGAACCGATAAG* |  |
| *MaPLDβ2-F* | *TCAAAAGGCATGATAGTGGATG* |  |
| *MaPLDβ2-R* | *ATTCAAGGCTTTCTGGTTGTGT* |  |
| *MaPLDβ3-F* | *GTGATACTGGGATCAGCAAACA* |  |
| *MaPLDβ3-R* | *CCACAGCGACATTCTATAACCA* |  |
| *MaPLDδ1-F* | *ATCCTAGTGGGAACTCAACGAA* |  |
| *MaPLDδ1-R* | *TGCTCATAAGTCTTCCCCTCAT* |  |
| *MaPLDδ2-F* | *TGTTCAGGTCTTCCGGTCTATT* |  |
| *MaPLDδ2-R* | *ATGTATATGAAGTGGCGTGCAG* |  |
| *MaPLDδ5-F* | *CCTGATAGCACGGAATGTGTAA* |  |
| *MaPLDδ5-R* | *ATCGGGAAAATACTCATGGTTG* |  |
| *MaDGK1-F* | *TAAAGCTTGAAGTCGATGGTCA* |  |
| *MaDGK1-R* | *CTACCTGAAGTTTCCCAAGGTG* |  |
| *MaDGK2-F* | *CTTGTCTGTGGTGGAGATGGTA* |  |
| *MaDGK2-R* | *CGATCTAGCATCGTAACTGCTG* |  |
| *MaDGK3-F* | *TAGGGCCATTGTTCTTCTGAAT* |  |
| *MaDGK3-R* | *CTAAGTTCAGCCATGACACAGG* |  |
| *MaPLC1-F* | *CTTTGATTCATATTCCCCTCCA* |  |
| *MaPLC1-R* | *ACTCTGATATGGGCAAGCAAAT* |  |
| *MaPLC2-F* | *GGCACAAGGATTTTAGTCGAAC* |  |
| *MaPLC2-R* | *TCCACCAAAGTCATCCATATCA* |  |
| *MaNAC25-F* | *CACTTCCACATTAATCCCCAAT* |  |
| *MaNAC25-R* | *ATCCTGGAAGTTGAGGCTATCA* |  |
| *MaNAC28-F* | *AGAGCTCGTGGGTTTCTATCTG* |  |
| *MaNAC28-R* | *TCCTGTACTTCCTACCCCTCAA* |  |
| *SlActin-F* | *GGGATGGAGAAGTTTGGTGGTGG* |  |
| *SlActin-R* | *CTTCGACCAAGGGATGGTGTAGC* |  |
| *SlPLDα1-F* | *GGAGCTATTGGTTTCAACAAGG* |  |
| *SlPLDα1-R* | *AAGCTCTTCCAATGACTTCTGC* |  |
| *SlPLDα2-F* | *ACCAGCAAGAGGTCAAATTCAT* |  |
| *SlPLDα2-R* | *GGGTATCGGAGTAAGTGACCAG* |  |
| *SlPLDβ1-F* | *AAACACATATGAACCCCAGGAC* |  |
| *SlPLDβ1-R* | *TTCCAGAGAACGTTGATTGATG* |  |
| *SlPLDβ2-F* | *ATATGTGAAGGCTATCCGTGCT* |  |
| *SlPLDβ2-R* | *CCTCTCATGCGCTCTTATCTTT* |  |
| *SlPLDδ3-F* | *CACATGGGCAAGTTAGACGATA* |  |
| *SlPLDδ3-R* | *ACTTGGACGGGATACTTGAGAA* |  |
| *SlPLDδ5-F* | *CGTATTTTCCAGTGAGGAAAGG* |  |
| *SlPLDδ5-R* | *TCAAGTGATGAGCTTCTGCAAT* |  |
| *SlDGK1-F* | *TTCTGCAGCTACTGTGAAGAGC* |  |
| *SlDGK1-R* | *TCCACCAGAGGATGTCCTAGTT* |  |
| *SlDGK2-F* | *CAGGAGGCTTATTCTCTCTCCA* |  |
| *SlDGK2-R* | *ACTGCCTTGCACTTTACCATTT* |  |
| *SlDGK3-F* | *GCTAGGTTGCAGGATCTTATGG* |  |
| *SlDGK3-R* | *GCCACGATCCTTATCTTTTCAC* |  |
| *SlDGK4-F* | *GGTCCAATTAACCGTCTGGATA* |  |
| *SlDGK4-R* | *GATGGAAGCCATAAGCTACCTG* |  |
| *SlDGK8-F* | *GCAAAGGTTGCATTAGAAATCC* |  |
| *SlDGK8-R* | *CTACTCCACCCATGTAGCTTCC* |  |
| *SlPLC1-F* | *CTGATGCAAATAATGAGGTCCA* |  |
| *SlPLC1-R* | *TGTCCTCTATTGCCTTGGTTTT* |  |
| *SlPLC2-F* | *TGAGTTTCCACTAACCGTTCCT* |  |
| *SlPLC2-R* | *TTTCCGATCATAGAGTGGGACT* |  |
| *SlPLC3-F* | *CAACTTGGGATGAACAGTTTGA* |  |
| *SlPLC3-R* | *TCGAAACGCATAAGTAGCTTGA* |  |
| *SlPLC4-F* | *TTCATGGAAGGACAGTGACAAC* |  |
| *SlPLC4-R* | *TCAGATTCAGGAACAAAGAGCA* |  |
| *SlPLC5-F* | *ATCTCCAGTGGGAAAAGATTCA* |  |
| *SlPLC5-R* | *TCCTCTCTTAGTGCACGTTTCA* |  |
| *MaNAC25-GFP-F* | *caaattcgcgaccggtATGGCTGATGTCTCCGTGGTCA* | Subcellular localization |
| *MaNAC25-GFP-R* | *tgctagtcataccggtGTACTGCCAGAAGTTCTCCAG* |  |
| *MaNAC28-GFP-F* | *caaattcgcgaccggtATGGAGGAGAGGAGGGAGAAGA* |  |
| *MaNAC28-GFP-R* | *tgctagtcataccggt**GGGGTATTGGCAACAGGAG* |  |
| *MaNAC25-GST-F* | *ggttccgcgtggatccATGGCTGATGTCTCCGTGGTCA* | EMSA |
| *MaNAC25-GST-R* | *agtcacgatgcggccgcTCAGTACTGCCAGAAGTTCTCCAG* |  |
| *MaNAC28-GST-F* | *ggttccgcgtggatccATGGAGGAGAGGAGGGAGAAGA* |  |
| *MaNAC28-GST-R* | *agtcacgatgcggccgcCTAGGGGTATTGGCAACAGGAG* |  |
| *MaPLDα1-MaNAC25-probe-F* | *TCGATCGTGGTTGTGCTACTTCTTGTTTGAAGCCTGATGAATCCAGCTTCGATTGTTTCACGTGAAGCTACCT* |  |
| *MaPLDα1-MaNAC25-probe-R* | *AGGTAGCTTCACGTGAAACAATCGAAGCTGGATTCATCAGGCTTCAAACAAGAAGTAGCACAACCACGATCGA* |  |
| *MaPLDα4-MaNAC25-probe-F* | *TTACGTGAAGTTAAGGATCACGTCGACCTTCACCGGTTGCAACCTCCCCTTGCCGATACGTACTTCGTGGCCTT* |  |
| *MaPLDα4-MaNAC25-probe-R* | *AAGGCCACGAAGTACGTATCGGCAAGGGGAGGTTGCAACCGGTGAAGGTCGACGTGATCCTTAACTTCACGTAA* |  |
| *MaPLDβ1-MaNAC25-probe-F* | *AATTGCACGAATATCAAAGCAGCACGGACGTCGAGCTCGTGTAGAAATGAATGTAACGCACGGTGGA* |  |
| *MaPLDβ1-MaNAC25-probe-R* | *TCCACCGTGCGTTACATTCATTTCTACACGAGCTCGACGTCCGTGCTGCTTTGATATTCGTGCAATT* |  |
| *MaPLDβ2-MaNAC25-probe-F* | *AGAACCACGGACGGAGGGACATGAGGCTCACCTGCACGGCGGAGGGTTTCAGCTCCGTGGCCGA* |  |
| *MaPLDβ2-MaNAC25-probe-R* | *TCGGCCACGGAGCTGAAACCCTCCGCCGTGCAGGTGAGCCTCATGTCCCTCCGTCCGTGGTTCT* |  |
| *MaPLDβ3-MaNAC25-probe-F* | *GAAGGCGTGGGCGACTCCGCCCTGCCGATTCGGACATCGCACCCGGGCTTTCTCGGCCGTCGTGGCGGCGTGCGGAT* |  |
| *MaPLDβ3-MaNAC25-probe-R* | *ATCCGCACGCCGCCACGACGGCCGAGAAAGCCCGGGTGCGATGTCCGAATCGGCAGGGCGGAGTCGCCCACGCCTTC* |  |
| *MaPLDδ1-MaNAC25-probe-F* | *GTGGGGCCCACGATGGCGTGTTGCTGGTAGGTGAGCTGGTCCGACGTGACCGGGT* |  |
| *MaPLDδ1-MaNAC25-probe-R* | *ACCCGGTCACGTCGGACCAGCTCACCTACCAGCAACACGCCATCGTGGGCCCCAC* |  |
| *MaPLDδ2-MaNAC25-probe-F* | *AGCCCGACGTGAAATCCCTGAAAAGTTTCAACGGTCATTCGAGTTTCAACTTTGACGGGTGT* |  |
| *MaPLDδ2-MaNAC25-probe-R* | *ACACCCGTCAAAGTTGAAACTCGAATGACCGTTGAAACTTTTCAGGGATTTCACGTCGGGCT* |  |
| *MaPLDδ5-MaNAC25-probe-F* | *AGATTCACGCAGCGTGACTGCTTTGCTCCGTCCTCGTGTGAGGAGGGGTTTGCGTGGACAG* |  |
| *MaPLDδ5-MaNAC25-probe-R* | *CTGTCCACGCAAACCCCTCCTCACACGAGGACGGAGCAAAGCAGTCACGCTGCGTGAATCT* |  |
| *MaDGK1-MaNAC25-probe-F* | *AAGTCGCACGTCCGCTACGCATGCGTTTATATACGTGTATATGGACATCGTGAGTGCG* |  |
| *MaDGK1-MaNAC25-probe-R* | *CGCACTCACGATGTCCATATACACGTATATAAACGCATGCGTAGCGGACGTGCGACTT* |  |
| *MaDGK2-MaNAC25-probe-F* | *TTTCTCGTGATTAAGCGTCTCAGGGCGGAAGTCTTTTTACATGAAATTGCTTACATTAAGCTTCGGTA* |  |
| *MaDGK2-MaNAC25-probe-R* | *TACCGAAGCTTAATGTAAGCAATTTCATGTAAAAAGACTTCCGCCCTGAGACGCTTAATCACGAGAAA* |  |
| *MaDGK3-MaNAC25-probe-F* | *TCTCGGCTTCGTGTGCTTCGCTTTCTTGGGGAAGACGGGACGCGAGTGCGTAAATAG* |  |
| *MaDGK3-MaNAC25-probe-R* | *CTATTTACGCACTCGCGTCCCGTCTTCCCCAAGAAAGCGAAGCACACGAAGCCGAGA* |  |
| *MaPLC1-MaNAC25-probe-F* | *ATCTCGACGAATAAATCCTTAACTCGTCAACGACTCGACTCGACTCGACTCGTGTCGGA* |  |
| *MaPLC1-MaNAC25-probe-R* | *TCCGACACGAGTCGAGTCGAGTCGAGTCGTTGACGAGTTAAGGATTTATTCGTCGAGAT* |  |
| *MaPLC2-MaNAC25-probe-F* | *AGAATGACGGGGCATCGCAAAGTGAGGAAGGGCACGCCTAACTCCAGTCGCATCCACGGCCGTCCAAGCGAAGA* |  |
| *MaPLC2-MaNAC25-probe-R* | *TCTTCGCTTGGACGGCCGTGGATGCGACTGGAGTTAGGCGTGCCCTTCCTCACTTTGCGATGCCCCGTCATTCT* |  |
| *MaNAC28-MaNAC25-probe-F* | *CTGGAGCTTCCGCTGCATGCAAGTTTACACTCGTCATCCTGTCAATAGCCACAGACACTCGACGCCCGAGGACGGACTT* |  |
| *MaNAC28-MaNAC25-probe-R* | *AAGTCCGTCCTCGGGCGTCGAGTGTCTGTGGCTATTGACAGGATGACGAGTGTAAACTTGCATGCAGCGGAAGCTCCAG* |  |
| *MaPLDα1-MaNAC28-probe-F* | *TCGATCGTGGTTGTGCTACTTCTTGTTTGAAGCCTGATGAATCCAGCTTCGATTGTTTCACGTGAAGCTACCT* |  |
| *MaPLDα1-MaNAC28-probe-R* | *AGGTAGCTTCACGTGAAACAATCGAAGCTGGATTCATCAGGCTTCAAACAAGAAGTAGCACAACCACGATCGA* |  |
| *MaPLDα4-MaNAC28-probe-F* | *TTACGTGAAGTTAAGGATCACGTCGACCTTCACCGGTTGCAACCTCCCCTTGCCGATACGTACTTCGTGGCCTT* |  |
| *MaPLDα4-MaNAC28-probe-R* | *AAGGCCACGAAGTACGTATCGGCAAGGGGAGGTTGCAACCGGTGAAGGTCGACGTGATCCTTAACTTCACGTAA* |  |
| *MaPLDβ1-MaNAC28-probe-F* | *AATTGCACGAATATCAAAGCAGCACGGACGTCGAGCTCGTGTAGAAATGAATGTAACGCACGGTGGA* |  |
| *MaPLDβ1-MaNAC28-probe-R* | *TCCACCGTGCGTTACATTCATTTCTACACGAGCTCGACGTCCGTGCTGCTTTGATATTCGTGCAATT* |  |
| *MaPLDβ2-MaNAC28-probe-F* | *AGAACCACGGACGGAGGGACATGAGGCTCACCTGCACGGCGGAGGGTTTCAGCTCCGTGGCCGA* |  |
| *MaPLDβ2-MaNAC28-probe-R* | *TCGGCCACGGAGCTGAAACCCTCCGCCGTGCAGGTGAGCCTCATGTCCCTCCGTCCGTGGTTCT* |  |
| *MaPLDβ3-MaNAC28-probe-F* | *GAAGGCGTGGGCGACTCCGCCCTGCCGATTCGGACATCGCACCCGGGCTTTCTCGGCCGTCGTGGCGGCGTGCGGAT* |  |
| *MaPLDβ3-MaNAC28-probe-R* | *ATCCGCACGCCGCCACGACGGCCGAGAAAGCCCGGGTGCGATGTCCGAATCGGCAGGGCGGAGTCGCCCACGCCTTC* |  |
| *MaPLDδ1-MaNAC28-probe-F* | *GTGGGGCCCACGATGGCGTGTTGCTGGTAGGTGAGCTGGTCCGACGTGACCGGGT* |  |
| *MaPLDδ1-MaNAC28-probe-R* | *ACCCGGTCACGTCGGACCAGCTCACCTACCAGCAACACGCCATCGTGGGCCCCAC* |  |
| *MaPLDδ2-MaNAC28-probe-F* | *AGCCCGACGTGAAATCCCTGAAAAGTTTCAACGGTCATTCGAGTTTCAACTTTGACGGGTGT* |  |
| *MaPLDδ2-MaNAC28-probe-R* | *ACACCCGTCAAAGTTGAAACTCGAATGACCGTTGAAACTTTTCAGGGATTTCACGTCGGGCT* |  |
| *MaPLDδ5-MaNAC28-probe-F* | *AGATTCACGCAGCGTGACTGCTTTGCTCCGTCCTCGTGTGAGGAGGGGTTTGCGTGGACAG* |  |
| *MaPLDδ5-MaNAC28-probe-R* | *CTGTCCACGCAAACCCCTCCTCACACGAGGACGGAGCAAAGCAGTCACGCTGCGTGAATCT* |  |
| *MaDGK1-MaNAC28-probe-F* | *AAGTCGCACGTCCGCTACGCATGCGTTTATATACGTGTATATGGACATCGTGAGTGCG* |  |
| *MaDGK1-MaNAC28-probe-R* | *CGCACTCACGATGTCCATATACACGTATATAAACGCATGCGTAGCGGACGTGCGACTT* |  |
| *MaDGK2-MaNAC28-probe-F* | *TTTCTCGTGATTAAGCGTCTCAGGGCGGAAGTCTTTTTACATGAAATTGCTTACATTAAGCTTCGGTA* |  |
| *MaDGK2-MaNAC28-probe-R* | *TACCGAAGCTTAATGTAAGCAATTTCATGTAAAAAGACTTCCGCCCTGAGACGCTTAATCACGAGAAA* |  |
| *MaDGK3-MaNAC28-probe-F* | *TCTCGGCTTCGTGTGCTTCGCTTTCTTGGGGAAGACGGGACGCGAGTGCGTAAATAG* |  |
| *MaDGK3-MaNAC28-probe-R* | *CTATTTACGCACTCGCGTCCCGTCTTCCCCAAGAAAGCGAAGCACACGAAGCCGAGA* |  |
| *MaPLC1-MaNAC28-probe-F* | *ATCTCGACGAATAAATCCTTAACTCGTCAACGACTCGACTCGACTCGACTCGTGTCGGA* |  |
| *MaPLC1-MaNAC28-probe-R* | *TCCGACACGAGTCGAGTCGAGTCGAGTCGTTGACGAGTTAAGGATTTATTCGTCGAGAT* |  |
| *MaPLC2-MaNAC28-probe-F* | *AGAATGACGGGGCATCGCAAAGTGAGGAAGGGCACGCCTAACTCCAGTCGCATCCACGGCCGTCCAAGCGAAGA* |  |
| *MaPLC2-MaNAC28-probe-R* | *TCTTCGCTTGGACGGCCGTGGATGCGACTGGAGTTAGGCGTGCCCTTCCTCACTTTGCGATGCCCCGTCATTCT* |  |
| *MaNAC25-MaNAC28-probe-F* | *CAGTCCACGGCACGGACCAGCTTCGACAAATTTATCCTTGCAAGTGGCTGAGTCTACACGCAGAA* |  |
| *MaNAC25-MaNAC28-probe-R* | *TTCTGCGTGTAGACTCAGCCACTTGCAAGGATAAATTTGTCGAAGCTGGTCCGTGCCGTGGACTG* |  |
| *MaPLDα1- qchip-F* | *TCGTGGTTGTGCTACTTCTTGT* |  |
| *MaPLDα1- qchip-R* | *CCGTTGAAACCTACACAATCAA* | ChIP-qPCR |
| *MaPLDα4- qchip-F* | *CCGGATCTTACTTTTTCTGTCG* |  |
| *MaPLDα4- qchip-R* | *GGTCGACGTGATCCTTAACTTC* |  |
| *MaPLDβ1- qchip-F* | *CGAGCTCGTGTAGAAATGAATG* |  |
| *MaPLDβ1- qchip-R* | *ACGTGAAGAGAAATGGCTTGAT* |  |
| *MaPLDβ2- qchip-F* | *GAGAAGCCAAAAATCAGAAACG* |  |
| *MaPLDβ2- qchip-R* | *AACCCTCTCCTCTTCCTCTTGT* |  |
| *MaPLDβ3- qchip-F* | *GATAGCAACAACCAGCGAACAC* |  |
| *MaPLDβ3- qchip-R* | *GATCGATGCAGAGAGTGGTTAC* |  |
| *MaPLDδ1- qchip-F* | *TTTTGATTGGAGGAGCAGTGGG* |  |
| *MaPLDδ1- qchip-R* | *GCTGGCCAGCAAAGAATTTGAG* |  |
| *MaPLDδ2- qchip-F* | *TGTACCGTTCATTGGAGAGATG* |  |
| *MaPLDδ2- qchip-R* | *GAAAGGATGCATCAAGAAGGTC* |  |
| *MaPLDδ5- qchip-F* | *GTGATGCGACATGGACACTTCT* |  |
| *MaPLDδ5- qchip-R* | *ACGAGTCATTCAAGTGGGATTT* |  |
| *MaDGK1- qchip-F* | *GTTTTACGAAGTCGCACGTCCG* |  |
| *MaDGK1- qchip-R* | *AAACCCCTCAATTCCTCTCCCG* |  |
| *MaDGK2- qchip-F* | *CTCTTCCTCTTCGTTTGCAGTT* |  |
| *MaDGK2- qchip-R* | *CAACGAATCCCCAAGGAAACCA* |  |
| *MaDGK3- qchip-F* | *ACCCTTTTGCCAGAGTTATTCC* |  |
| *MaDGK3- qchip-R* | *CTCGCTCCATGCCACTATTTAC* |  |
| *MaPLC1- qchip-F* | *ATCCCACTGAATGTTTTGAGGA* |  |
| *MaPLC1- qchip-R* | *AATCTGAAAAAGGGGGCATCT* |  |
| *MaPLC2- qchip-F* | *GTCGATTGGAATTCTTCGTCTC* |  |
| *MaPLC2- qchip-R* | *TTTGGGCACAGTTTCTATTTCC* |  |
| *MaNAC28- qchip-F* | *CTCATGGGAAAGGAACAGATTC* |  |
| *MaNAC28- qchip-R* | *GCTATTGACAGGATGACGAGTG* |  |
| *MaNAC25- qchip-F* | *CACCATTATTGCCTTCACAAGA* |  |
| *MaNAC25- qchip-R* | *TGCACCACTTGGTTGTTTTTAC* |  |
| *MaNAC25-PBD -F* | *tcgccgaccggtaggcctATGGCTGATGTCTCCGTGGTCA* | Transient expression assay |
| *MaNAC25-PBD -R* | *aaccagagttaaaggcctTCAGTACTGCCAGAAGTTCTCCAG* |  |
| *MaNAC28-PBD -F* | *tcgccgaccggtaggcctATGGAGGAGAGGAGGGAGAAGA* |  |
| *MaNAC28-PBD -R* | *aaccagagttaaaggcctCTAGGGGTATTGGCAACAGGAG* |  |
| *MaNAC25-PEAQ -F* | *caaattcgcgaccggtATGGCTGATGTCTCCGTGGTCA* |  |
| *MaNAC25-PEAQ -R* | *agttaaaggcctcgagTCAGTACTGCCAGAAGTTCTCCAG* |  |
| *MaNAC28-PEAQ -F* | *caaattcgcgaccggtATGGAGGAGAGGAGGGAGAAGA* |  |
| *MaNAC28-PEAQ -R* | *agttaaaggcctcgagCTAGGGGTATTGGCAACAGGAG* |  |
| *MaPLDα1-0800-F* | *tatagggcgaattgggtaccCCTTACTCTATGTCATGGACCCG* |  |
| *MaPLDα1-0800-R* | *ttggcgtcttccatggCCGTTACCTTGTTACCCAGTTCCAG* |  |
| *MaPLDα4-0800-F* | *tatagggcgaattgggtaccGCCCAAGCAATTTCACTCTCTGGTC* |  |
| *MaPLDα4-0800-R* | *ttggcgtcttccatggCCCTTTGTCTTCTTCTTACTGTTGCTGATT* |  |
| *MaPLDβ1-0800-F* | *tatagggcgaattgggtaccTACAGCATTAAAACAAGGGTATCAAATA* |  |
| *MaPLDβ1-0800-R* | *ttggcgtcttccatggGGATTCGTTCCTTCCTCTGTTTCGCTCAG* |  |
| *MaPLDβ2-0800-F* | *tatagggcgaattgggtaccTTGTCCCTTTTGCACAGTGGCCAAAGG* |  |
| *MaPLDβ2-0800-R* | *ttggcgtcttccatggCGTGACCGGAGGATAGAGAGGGGCGTAG* |  |
| *MaPLDβ3-0800-F* | *tatagggcgaattgggtaccATGATAGCAACAACCAGCGAACACGTGGGC* |  |
| *MaPLDβ3-0800-R* | *ttggcgtcttccatggCGACTTCTTCTTCCTCTGCTTCTCCCAC* |  |
| *MaPLDδ1-0800-F* | *tatagggcgaattgggtaccTCTGCTTTGCCCGCCTCCTAC* |  |
| *MaPLDδ1-0800-R* | *ttggcgtcttccatggTCGATCCAAATCATCAATTCAATCACTAGTCATT* |  |
| *MaPLDδ2-0800-F* | *tatagggcgaattgggtaccCCGAATATGTAATCATTGCCACCATGCAGTCAAGCTG* |  |
| *MaPLDδ2-0800-R* | *ttggcgtcttccatggCGCAGTCGAGTTGTGGGTAACTTG* |  |
| *MaPLDδ5-0800-F* | *tatagggcgaattgggtaccCGCCTGTGGATTCCATATTTGGTCCAC* |  |
| *MaPLDδ5-0800-R* | *ttggcgtcttccatggCGCGCGCTCCGGTGAAGGCCGAGGGAG* |  |
| *MaDGK1-0800-F* | *tatagggcgaattgggtaccCTTTGTTTTACGAAGTCGCACGTCCGCTACGCATG* |  |
| *MaDGK1-0800-R* | *ttggcgtcttccatggAATTATTAGCCTCAGTTGGATCCAAAAAAAAATCTG* |  |
| *MaDGK2-0800-F* | *tatagggcgaattgggtaccGGAAATATTCCCGGGTTGACCTG* |  |
| *MaDGK2-0800-R* | *ttggcgtcttccatggTATTAGCCTCAGTTGCTCCACTCATCA* |  |
| *MaDGK3-0800-F* | *tatagggcgaattgggtaccTCGATTATTGCCTCAAGACAGCACG* |  |
| *MaDGK3-0800-R* | *ttggcgtcttccatggCGCTACCTTTCGCTCGCTCCAT* |  |
| *MaPLC1-0800-F* | *tatagggcgaattgggtaccGATGCATTTGGCAGCTGGAAGAGTG* |  |
| *MaPLC1-0800-R* | *ttggcgtcttccatggTCCGCGATTCCACCTCCTCCCT* |  |
| *MaPLC2-0800-F* | *tatagggcgaattgggtaccGGAGTCTCAAGTGAGAATCGCATGACGCT* |  |
| *MaPLC2-0800-R* | *ttggcgtcttccatggCGCTCCGCGCCGTCAGCCGGCGTCCCCC* |  |
| *MaNAC25-0800-F* | *tatagggcgaattgggtaccTGCTATGTTGCTGGAGTTGTGTGACATGG* |  |
| *MaNAC25-0800-R* | *ttggcgtcttccatggGGTTGCACCTCTAGACACAAAGCAAAGAA* |  |
| *MaNAC28-0800-F* | *tatagggcgaattgggtaccCTTTGACCACACTTAGATGCGGTGTCCAT* |  |
| *MaNAC28-0800-R* | *ttggcgtcttccatggGATTATTCTTCCTTTCTTCTACTCCCCC* |  |
| *MaNAC25-DBD-F* | *catggaggccgaattcATGGCTGATGTCTCCGTGGTCA* | Y2H analysis |
| *MaNAC25-DBD-R* | *gccgctgcaggtcgacgTCAGTACTGCCAGAAGTTCTCCAG* |  |
| *MaNAC28-DBD-F* | *catggaggccgaattcATGGAGGAGAGGAGGGAGAAGA* |  |
| *MaNAC28-DBD-R* | *gccgctgcaggtcgacgCTAGGGGTATTGGCAACAGGAG* |  |
| *NAC25X-pLP100-F* | *tttggagaggacagggtacccgggATGGCTGATGTCTCCGTGGTCA* | Genetic Transformation |
| *NAC25X-pLP100-R* | *ggtcgactctagaggatccccgggGTACTGCCAGAAGTTCTCCAG* |  |
| *NAC28X-pLP100-F* | *tttggagaggacagggtacccgggATGGAGGAGAGGAGGGAGAAGA* |  |
| *NAC28X-pLP100-R* | *ggtcgactctagaggatccccgggGGGGTATTGGCAACAGGAG* |  |

**Table S2. Basic information of MaNAC25 and MaNAC28 proteins.**

| Protein name | The open reading frame (ORF)  (bp) | Amino acid (aa) | Molecular weight (kDa) | Isoelectric point (pI) |
| --- | --- | --- | --- | --- |
| MaNAC25 | 1038 | 346 | 84.96 | 5.04 |
| MaNAC28 | 912 | 304 | 73.1 | 5.08 |

**Table S3. Id percentage and score information obtained by comparising *MaPLDs*, *MaDGKs*, *MaPLCs* from different subgroups selected in banana with *PLDs*, *DGKs*, *PLCs* in tomato database.**

| **Group** | **Protein name** | **Subject Id** | **id%** | | | |  | **Score** | | | |
| --- | --- | --- | --- | --- | --- | --- | --- | --- | --- | --- | --- |
|  |  |  | MaDGK1 | | MaDGK2 | |  | MaDGK1 | | MaDGK2 | |
| Cluster Ⅰ | SlDGK2 | Solyc05g056420.3 | 62.24 | | 63.95 | |  | 926 | | 953 | |
|  | SlDGK1 | Solyc12g039180.2 | 51.71 | | 52.05 | |  | 755 | | 751 | |
|  | SlDGK8 | Solyc03g115370.3 | 52.97 | | 52.76 | |  | 726 | | 726 | |
|  |  |  | MaDGK3 | | | |  | MaDGK3 | | | |
| Cluster Ⅱ | SlDGK3 | Solyc07g006580.3 | 52.74 | | | |  | 509 | | | |
|  | SlDGK4 | Solyc06g009270.3 | 51.38 | | | |  | 504 | | | |
|  |  |  | MaPLDα1 | | MaPLDα4 | |  | MaPLDα1 | | MaPLDα4 | |
| α | SlPLDα2 | Solyc03g116620.3 | 45.45 | | 48.73 | |  | 251 | | 770 | |
|  | SlPLDα1 | Solyc06g068090.3 | 45.14 | | 48.41 | |  | 249 | | 760 | |
|  |  |  | MaPLDβ1 | MaPLDβ2 | | MaPLDβ3 |  | MaPLDβ1 | MaPLDβ2 | | MaPLDβ3 |
| β | SlPLDβ2 | Solyc01g091910.4 | 69.56 | 69.34 | | 66.74 |  | 1281 | 1244 | | 1264 |
|  | SlPLDβ1 | Solyc08g080130.3 | 69.6 | 66.83 | | 69.73 |  | 1244 | 1197 | | 1235 |
|  |  |  | MaPLDδ1 | MaPLDδ2 | | MaPLDδ5 |  | MaPLDδ1 | MaPLDδ2 | | MaPLDδ5 |
| δ | SlPLDδ3 | Solyc04g082000.3 | 63.7 | 61.73 | | 67 |  | 1170 | 1108 | | 1224 |
|  | SlPLDδ5 | Solyc02g061850.3 | 61.41 | 59.56 | | 64.92 |  | 1117 | 1059 | | 1192 |
|  |  |  | MaPLC1 | | MaPLC2 | |  | MaPLC1 | | MaPLC2 | |
| NPC | SlPLC4 | Solyc06g007120.3 | 65.77 | | 62.27 | |  | 827 | | 776 | |
|  | SlPLC1 | Solyc10g076710.2 | 61.27 | | 55.17 | |  | 742 | | 674 | |
|  | SlPLC3 | Solyc05g052760.3 | 61.74 | | 59.32 | |  | 739 | | 710 | |
|  | SlPLC5 | Solyc06g051630.4 | 59.87 | | 59.63 | |  | 736 | | 729 | |
|  | SlPLC2 | Solyc06g051620.3 | 57.38 | | 58.7 | |  | 696 | | 690 | |

**Text S1. Probes in promoters of *MaPLDα1/4*, *MaPLDβ1/2/3*, *MaPLDδ1/2/5*, *MaDGK1/2/3*, *MaPLC1/2*, *MaNAC25* and *MaNAC28* containing CA(A/C)G(T/C)(T/C/A)(T/C/A), (T/A)NN(C/T)(T/C/G)TNNNNNNNA(A/C)GN(A/C/T)(A/T), CATGTG, CACG, CGTA, GCTT from banana genome. The sequence of NAC binding site was indicated in red, and the probe used for EMSA was marked by box.**

>*MaPLDα1* (Ma10_g26900) promoter

CCTTACTCTATGTCATGGACCCGGCATCTTTAGGACTCGGGGACGACGACTGGAGGAGAGAGAAATATATTTTACACAATGTGATTATTATCGATCAACCACATGACCTTCGCAATGTTGCATCCTCATTTCCGCTGTATTTTATACTGGTCAGAGTATGGTCTTTTTCTAGTGTCTAAAATTTAGATGGTATTTTTATATTGATCCTATCCAGAGCTACTATTAACAATAGATAAAGAAATGTGATGTCCTTCTCCAGTTGCTTAACACACTTGTTTTCTTTTTATCTTTTTTTTTTTGGGTTCTTTTCATTGCCTGTATCAGAAAGAATTTGCTATACCTTCAGAAATATAATAATAACGTATATTCCACTAAAGAACATCAGCTGGACAATTGTATTTGTTTGCCCTTTAGTTATATATGGTGGAATTTGTTACTCTGTTTTCTCAAAATTGAAAAGACTTGTAAAAGGAAAGCTTATAAAAGGTTTAGAAAACACATAAAGATGCCATAACTTTTTGTAGTCCTTTGTCTTTATAATGACTTGATGAAGGAAAGTTGATGTTGAAGTCAACTCATCGTAGTTCGAGGTGACTGGCTTGATTTGGGAAGGGTTGATCTTGGAAGAATTTGGATTTGGAATATTGAGATTGATAAAAGTTAGTTAAGTTGACGTGTATTTGACCTGTCAAAGTCTAATTATTTTAAGGAATCTTACATATATATCATTGTAAAATTTTAAATAGCATATACTATTACAAATTTTAATGTAGCTTATATCTTCCTCCACAGCTATTACCATCCATCCAAAAAAGAGTTAATTTTATTTGAGTTTATAAAATACATAAAATATTTTCTATTTATATTAATGAGTTTTCACAGAATATTTGGTATAAATAAGAATATTTGGGGATTATTTATATATATATATAGGCGTTAGGTGATATTTTAAAGTCTTGAGGGACCAATATGAAATTTTGATAAGAGCAGAGTGATGTATACAAAATCTACCTTTTTGTTATTTCTTTTATTTGTTAATATCACATTGTCATCCTTCCCACTTTCCATAGGCTCTTCTCAATTCCCGCTACTCGTCCCTCTTGATGTATTCACTACGCTTTTTCTATATATATGAGACCTAAACTAGCATTGAGAGGCAGTGAACTCTCTTCCTCCATTTCTCCCTCCATCTCCTTACCCTTCGCCTCCGATTTATCACTTGCTCCGCCGATCCAGGTGATGTTCCCTCGTCAGGTGAGTATAATGGAGGAATCCCCCTTTGCTCGATCGTGGTTGTGCTACTTCTTGTTTGAAGCCTGATGAATCCAGCTTCGATTGTTTCACGTGAAGCTACCTCGGATCCATCTCGTTCGATGACTTCTTTTTCGTTTTTCATTTTCGATTGTTCTTGAGCTAAATTGGATCCTTGATGTAGAACCATGACGCTCTTGCACTTGATTTCCTACTTGTATGGTTCGACTTTGACTCGGCCATCAAATTGGTTGATTGTGTAGGTTTCAACGGTGTGCCGTGGGAGGCAGATCCGATCGTTCGGTGACTCCGTTATTGAAGGTGACATCTTTTTTCCTTCATTTCTGCTATTTGCATCAGCTCGGTAGGACTTCAAATCTGTTTCCTCTGATGTTATTCTATTTATGTGTGTACACAGTCTGGAACTGGGTAACAAGGTAACGGATG

>*MaPLDα4* (Ma10_g23910) promoter

GCCCAAGCAATTTCACTCTCTGGTCGGTCGATGTCCCTCTCGCCTTTACCAATTACACATCCTCATCTTTAGTACAACACAATAATTAAAATAAGGAACTAATTAGACGATGTCACATCATTTCTTAATGTTTCAGGAACATTTATGTACGACACGATGAACCCACGAGGAACCTACTGTCGCGCACCTTCTCTACCTTCCTTGCCTGATGTCAAGGTCACTGGTTTTGCAGGTGAACAGTCGTCGCCGGTCGTGGGGAGGTGGGTTAGCTACGGTTGTCACCGTCTCTATCGCCTCAAGAACGCTATCGATGGCGTCGCCGTCGTCATTGCTCAGCCATGGCGCCACCTGCCGACGACTGACATAACGCGTGCAGTGCGAGTGGGCAATCGCATTTTCAACGAGGACTCGTTCCATTCGTCTATCCTTTTCACTATCATTATTATCCGTCTCCTTCTTTCAATTCACCGCTCTCTTCTTTCTTATGATCATGTTTTCTTCGATCGTGTCTGAATTTTCTTGATAGAATGAGTCATCGTTTGACTCGATCAAACTGGATTACCGTATCTCTTTCGTGGAACTTCATCTTGCTCATACTCATTTTCTTTCATCTTACCATTCAATCAGTCCCTTGTTTCATTAGGACTCTTCTTCCAACCCGGATCTTACTTTTTCTGTCGAAAAACTAAATCGAAATTCTATTCATTATTCCCAATATCTTCATGTGTTTCATGCAAACTCCAGCTTAATTTGAACATGCGTTGCTGCAATTCAAGTATCTTATCCACCCATGCATGCACGTTTCCCAGACGAAGCAATAGCAATCTGAATAGGAAAAGAACACAGAAGAATTTACGTGAAGTTAAGGATCACGTCGACCTTCACCGGTTGCAACCTCCCCTTGCCGATACGTACTTCGTGGCCTTTGGTTCCCATTGGTTCACCATCTTGTGGCCTCCCTTACATCGTTCCACTGGATAGCATCCATATTACCACTCTCCGCCGAGAATATATGTCCGAACACTAACGTACATACAATTACTATTATGATTATTATTCAAGTGCTTACTATTCAACTAACTTACAAATTCTAACTTAGTCATCTACGTGAGGCACATGTACAAGAATGAGGAAGTTGTAGAGGTCAGGCTATATATGTGTTCGTGGATAGGATCTTTGAGGCAATCAGCAACAGTAAGAAGAAGACAAAGGGATG

>*MaPLDβ1* (Ma05_g22560) promoter

TACAGCATTAAAACAAGGGTATCAAATATTGTGGGTTTGCCTTTAATTAATGGTTTTCAGCTTTCTATATAATTTTTATTCCCTCAAATATTGTGGGTATGGATCATCTCTTCCATCGTCATACTAATTTTTTATTATAATTTTAATTTTATTAAATATTTTAAAAAATATTCAGATTAATTAATGCATTATTAAACAAGTAATGAATCTTAAGTTCAAATTCACATGCTAACGTTATGTATATTATTTAACATCATAAAAATATATTTTTTAAATTTATCATTTAATGTAAAAATAATATATTTTAATTTCTAAAAATAAAAAACATGTCAGGTGTGGTATCTTGTGACTTGAGAGGCACAAATCCTATACTATTATGTTTGTAAATAATCCCTACAACTTTTAATCATGGTAGGCTAATTGCACGAATATCAAAGCAGCACGGACGTCGAGCTCGTGTAGAAATGAATGTAACGCACGGTGGAGGCTTGTCGGTTGGAGTGCACATGCATCGACCATGGTCTCCCGGCTCCCGCTATCCTCTCTGTCCATCAGCTCAACGGCAGAAACGTAGTCTTAGCCCACACCTCCGCTCCATCGCCAATCGCGCGATTTCAATCAAGCCATTTCTCTTCACGTCTTTTCCTATCGTTCGTTACTTTTCTTCTTCATCTCGATCCTAAAATTATCAGGAACCGTCTGACTTCTCTTCTTCATCCTCTTCTCGATCTCTGGATCTGGAACAGACACCAATGGAACAGAACAGAGCGTGGTGAAAGGGGCTGAGCGAAACAGAGGAAGGAACGAATCCATG

>*MaPLDβ2* (Ma04_g31450) promoter

TTGTCCCTTTTGCACAGTGGCCAAAGGCAATCATACAATTTTGCACTACTTCAGGTGGAAAGAAGCATTAGTCATAGACTGAGATACAAATATTCCTAGATTATATTCACAGTTTAGTCCTAAGAATTACCTTCAGTACCTGAAAAAGAATCAGGTCAGTACAAAACTCTAACAATCATCTCCCAGAAGACCAAGTTTTCAAGCATGAAATCTCTTGAATTTCAAATGGTGGCATCACAAGCAAAGCATTTTGCGCAACAAAATACAATCTTTAGGAACTTCCCCTCGCAAAAAGAACCACTCAAATACTAGGACCACCATAAAAATGAAGTCAAGGTAGAGACAAAGAAAAAAGGGTAGTTGACGAGCCAAAAGAGACTTGCTTACCATAAGCTCCGGCAAAACCCCATCTTGCTGCAAGAGAAGCCAAAAATCAGAAACGATGAACCGAATTGAGCACGAGCATCGATGCCCGGTACTGGAGAACCACGGACGGAGGGACATGAGGCTCACCTGCACGGCGGAGGGTTTCAGCTCCGTGGCCGAGGAATCCATGCGGAGGGCGGCGAAGGGGAGCCTCCGGGTCGCCGATGCGGTGCGAAGAGAGATGGGGAGCTTGGTCGGGAAGGGAGAGTAAGAACAAGAGGAAGAGGAGAGGGTTAACGCTGCGATCGCTACGGGGAGAGAAGAGACGCTTAAGATTTTGAGAACAGCTACCATTTTTCGTCCTCTTAATGAGGGTTTGGTTTCGCACTCCCTTCTCCGATTCACTCTTTTATTACTTGCTTTATAATTCTTTTTCCTAAAGCTTTTTATCCTATTCACTATCCCGTATTATATTGTGGAGAGTATAATTTTTGTGGAGTTTCTTTTTTTATTATTATAATCCTTCTTTTATTCGGCCCTTTGCAGAAGGTTCTGGAAGTATCTTGAGAAATCCAGCGGCCCGGTAGCTCGAAACCTAGACCGCGCTTCTCAATTGCGACGTACGTGGATTGTAATTGGAGGCAGGATTGGGTCCCACCTCGAGTGTTCCGGTTCCTGCGATAATGGCCATCATCGGGCTTTCACGAATCGCAATGGCGGGTTTTATAAAATCAGAGAACAGTGATGAGATCGATTGATCGGCGGTCGGGATTGTCCGAGAGGTACGAGCCGAGGAGAGCCCACTGCGGCCTTCTTGACGACGTAAACGGGCCCACAACGACCATCGAATCAACGACCTCGTTCTCCGCCTCGTCCACTTTTCCAAACCGTTAATTCTCCACACCCCCCCCGCCCCTCGCCTCCCTCTTTCCACCTTCCCCTCATCCTCGTTCTTTCGCCTCCGAGTCTCACCGGTTTCTCCTCCGTCCTCGGCGAAACCCATCGAGAAGCAGAGCTTTGCGTTCAAAGATACAGAGGTTTCATCTCTCTCTCTCTCTCTCTCCATGGATAACCACAGCAAGCCGCACCACCCATATCCTGACGATCATCAAACCCCCTACCAATACCCACCGCCTCGCCAGCTCCTACCTTCGAACTCTTTCTCCCATCCGTCGCCCGTATCATCGCCCCAATCCCTCGACCACTCTGGCCCGTTGCGTCACCCCCTGCCGCCGCCCTATTCCCTTTACTACGCCCCTCTCTATCCTCCGGTCACGATG

>*MaPLDβ3* (Ma08_g11770) promoter

ATGATAGCAACAACCAGCGAACACGTGGGCTTGGTCCTATCGTGCCTTTAATCAATCTGCCCATCTTTTCGTACAATATATATCAACCCGTCACGTTCCCCGTAACCACTCTCTGCATCGATCCACCGGACGCTCACCAGCTTGTCATCCCATCTGTGTGCCCAATGGACTCGTGCTAACCACGTGTCGTATAATAGAGACATGTATAGGAATTGCGGACCCCAGAGAGTATATTGATTTAAGTAGGACGATTTCTATAGAAAATATCTACATTTAGAATATTTTTCAATTAGTATATCTCATTTTATTTTTAAATAGTATCTTTTGTTTAAAAAAAACTTAAATTACTTCTTAAAAATATTTTCACCAATGTTTTGACTTGTTACAATGCTTTGACCATCATAATAAAAATACTTTAAAATTTACTTGAAAATACTATAAATGATATAAATAGACTTCTAACCTTAATCAAATTAATTATAAATTTAAAAATATAATATTATAATTATTAATAAGTAATGACAACTAAAGAGATATGTATAAAATCACTTATAGTATTTTTAATAATATTAATAATATTTTTAGTTAATTAATAAAAAATACTATAAATATTATTAAAAATTAATATATATGAGTTAAATAAAAAATATTATAATAATAAAAAATGATAAAAAAATTAATGAAAAAACTTGTAAAGAATATTTTAGGTATTTTTAAATAAAAATATTATTTAAAAAAATAAAAAAAAGAAAATATTTTTTTTTAAAATCCAAAATATAGATATTAGGATGACTACGGTAAGTAGCATCCGTCGGTAAACATCCGTCTTCTGACTCTCGTCTGGTTAAAGGCCACGTAAACATCTGCCACTGTTCTCGGCGAATCGATTCACCCGACGGCTTTTTCTTGTCGGTTCTTGAGTGGCGAAAAGTCGTGATCGCCGGGTGAAGGCGTGGGCGACTCCGCCCTGCCGATTCGGACATCGCACCCGGGCTTTCTCGGCCGTCGTGGCGGCGTGCGGATGAATAAAGATCATCGCGTTCAAGACCAACTCCAATTCGTCGTGCCTTCCCACCTTCCCTTCTTCTCGAGGAGGAGAAGGTAAACTCACGCCATCTTCTCAGCCACCGACTTTCCTCTTGCTTCTTCCTCACGCGTAAATTGATTCAGACCGGCGTCTCCTCGCCCTGTTTCTTCTGGCCCAAGAAAGCGATCAAGAAGCCGGAGCAGCAGAGCACTGCGTGGTGGGAGAAGCAGAGGAAGAAGAAGTCGATG

>*MaPLDδ1* (Ma07_g24920) promoter

TCTGCTTTGCCCGCCTCCTACCTTTCTTTTGATTGGAGGAGCAGTGGGGCCCACGATGGCGTGTTGCTGGTAGGTGAGCTGGTCCGACGTGACCGGGTAAGTAAGTGGTCTATGCATAAGGGATCGATTTGAGCGATGGGGATTCCCGCCTCCCTTGTAGCCGAAGTAACCGCTCCACCAACGGCAAAGCGGCCTCGTCACCGTCGTGAAACACCGGTCTTTATTTAGTCTCTCTCTCTCTCTCTCAAATTCTTTGCTGGCCAGCTCCGGTGTCGATTACTCAGGTATTCTCTCCCCGCGCCATGCTGATTTACTTACGAAACGTCAAGATCTTGGCGAAACCATAAGCTGCCAAGCCGGGAAAAATACAACCTTCTTACTGACCAAGCAGGTTCACGTTCAATTGTGATGTTGTTGAGCTTTATCTGCTTTGATCCCTCGATGTTCTTTACAGTTTCCTTCTTGAAATCTATTTATGCTTCATTTTCCGTTCAGTTTGTGACCAATTTCATCTTATTTAGTTGTTAATGGTTCATCTTTCCGTAATTTTCTTTGGTTAATGGCACAATTTTTGAAGTTGGTTTTAAACGGTATGAACGGTGGATGATTCCTTATGGTCTCAGTTGTTCTGAATTATCGTAATGCACTTTGTATTTGAGCAATGTTGTTTCTCATCAATGTAAATTGGTTCGTTGCACCAACTTGGATCTTGAATGATATACTGACCTTCCTTTCTTTGTATTTGGGCAATGCTGTTTCTCTCAGCTGCTGGCATTGGCGTTGCTGGTCATGCAAAGTTGGGTCACCTCTACATTGATTTTTGTCCTTGTATGGAAAAGACAACTTTTTAAAAATTGTTTAACATAATTTTCAAGCCCTGTTCCTGTGGATGCTTAATGTGTGAAGATTTTTATTTGCATTGATCAGATTTGAGTCAGCGGAGCGTGGACTGGTAACAAAGGTTTTACTTCCAAACTTGGAACTGCTAAGAAGATAAACTTCGGTTGACACATTCATTCATTTAGTTTTGGTTTTACGAAGGTTGCAAAGAAGAAGATAATTCTTCATATGCATGCCTGGTTCTTAAAACCTGCTGCATTTGAGGATTGAAAAGTACAAGGTACTTGGTAAAGCATCAACATATACACAACTATGGTTTATTCATCCATCTTGTGGTTCGTTTTCAGTAAAAAAGTGAAGTATGTGCAGGTGGTGGTGGCTTACCGTGAAATCACTCTCAATGACTAGTGATTGAATTGATGATTTGGACCGAATG

>*MaPLDδ2* (Ma11_g01110) promoter

CCGAATATGTAATCATTGCCACCATGCAGTCAAGCTGATATCTGGTTTTAGCGGACGAGAGATTATTGTTGGTGACACAAAGATCTATCATCACTTCTCCGATGGCACATGTAGTTGTGGTGATTATTGGTGACCTCAATCACGACATATATGTAGTAAAGATAAATTGAGTGCACTAAGGCATGATGTATACAGTACAAGTTAGCATGTATGTAAATAACGAAAGATGGAGGTGACATGCTATTTAAAGGAACCGAAAACTTGTCTCAGACAGAAGCTGTGATAATAACTTGTATTTGCAGCCGCCTGCAGTTGTTTATACACAGTGAGGGAGACAATCGAAGTGCCGCTTTTGTCAGTTTTCAATACAAAATTTGCATCTAACCTCCGATGATGAATCACCACCATTGCCATTCTTCTCAAAGATGTTCATTGTTAGTGTTGTCTTTGACAGGCTATAGAGGTTGTATGTAATCATATATGCTTCCATGAGTGAGGCGGAGGCCGTGTCAGAGGGCATCGACATGGAGCGACAGCGCAGTCGAGGGCAAGGTGTCGGCGGCGCGGGTGAGGGAAGCGCCGAGGACCGAAGCGGTCGGAGTATTGAACGATGTCATTCGGGTCGTCTGTTCCATCGGAACCGTCTGATTTCGGTAAGATCATGAGGAAGTGGATGCCTATTTTAGAAAGTAACGGTCGGATCGGGATTAGATCTGTACCGTTCATTGGAGAGATGATCCATGTATTGAACGGCAGAGGATCTCAGCCCGACGTGAAATCCCTGAAAAGTTTCAACGGTCATTCGAGTTTCAACTTTGACGGGTGTCCTCATGGCTTTTTGTGTTTTTTCCTTCAAAGCGAGAAGAGGAATACCGTTCTGATGGAAGCCACGTCGACCTTCTTGATGCATCCTTTCCTATTTCACGTTCAACAACTATCGAGCCCGCCCTTTCTCTTTGATCGGCAACCACTTCTTTCCGGTCCCCGACCTTCTTCAAGAACCTCTTGCTTCAAGAAAAGAACCTAATCCTTCCCTTCTTCTCCACTTCTCTCTCCCTCCTCTCTCTCTCTCTCTCTCTCTCTCTCTCTGTTATACTCCCCCTCGCTTCCGATGGCTCTTCCTCCTCCTCTTCTTGATCTTCCAAGTTACCCACAACTCGACTGCGATG

>*MaPLDδ5*(Ma07_g18790) promoter

CGCCTGTGGATTCCATATTTGGTCCACAGGGCATGGGCGAAAAAATGAACCATTAATTAGAAGAAAGAGTAATATTGATTTTAATATTCGTCTGCACATGGAACATTCATGTTCTGATGGTGATGCGACATGGACACTTCTGACGGAGCAAGTGCGACGCCAGGCTGTGGTAGCGTGGCATCGACGAGACGCTGAGATCCGCGGTCGGTGCGTGCCCACCCAATGGCCGGCCGTTGCGACCGCGTCGGTCGGACGTGCGTGGAACGTGCCAATATATATAATGATTCTTCGTATCTGTCAATGTAGCGTTTATACAATATCTGAACCTCTTAAGGTAAATCCCACTTGAATGACTCGTAAATGATTGAATGGCTATGTATATAAATAATATATATCTGCTATGTTAGAAGATTAAGATAATCGGACAAATGTTTTAGTGTAATGCTCGTCGATGAATTGTATTATATTGAAATTAAGATGATAATAAATTTATGTCATATGATATTAGTTTGACTTTTATGTTTTCCCACCTCATCTCAACTATTCTTACACCCGAAGCACACCTGACTTGTGATTGGCTCTCAACTCGTAGAGCCCGAGGAGGCATGCCTAATGAATGATTAAGGCACGGCACGTAACGTCAACGAGACCAAAAACCAATAATATCTGTAGGTCCAATCGTTGACGAAAGCGAAAACATCAATCAAAATGGCTTTGAGATTCACGCAGCGTGACTGCTTTGCTCCGTCCTCGTGTGAGGAGGGGTTTGCGTGGACAGCATCAATCAAACCCGGTAAGGAGAATTAAGGGATTTCGTAGCTAGCAAACTTGCTCCTACGTTTAAGCTCGACTTCTCGCGTCCCGACTTCGGCTCGCTCTTGGAAGACCGCCCCCCCCCCACCCATTCTCAGCTTCTATTTGGCATAGTCCATCCCGCGTTTGTCTCCCTCCCTCGGCCTTCACCGGAGCGCGCGATG

>*MaDGK1*(Ma02_g22740) promoter

CTTTGTTTTACGAAGTCGCACGTCCGCTACGCATGCGTTTATATACGTGTATATGGACATCGTGAGTGCGCGCATCGTATCCCATGTGATGCAACGATATAAAGCCGTCGACGGCCAAACCCGCCTTGGTTCCGTCGCCATTTCTCCTTTCCTTTCGCTTCTTCTTCTTCTTTCCTTCGCCTCTAAGGAAGAGGCGATCGAGAAAGAGAAGAGAGCGATAGAGTGCGGGAGAGGAATTGAGGGGTTTGGACTTTGGAGGTACGTAATTGGTTGCCTGTGTTTGCAGTTTTTTTGTTCTGGTAATGTAGGATCAGATCTGGCCCCGATGTTATGGGCATTCGCTGCCGCGGTGTCGGTTTATGTGTTGGCTTGATGATTCTTGAATCGCTGTTAGGGTTTTTGCTGTCTTTCGTCGGATGTAGCTTCGGTAGATTGATTTGGGCTTTTGACTGGCGTTTCCTTCTTGTTTTTGGACTTCTGCGTCTTCTTCTGCTTGTCGTTTCTGAGGTGCTCTCGTGATGGGACTTCCGTTTTGACATGAAGCTTCCATAGCTTAAAATTGATTCGCCTGTCGTAATAATGACAAAAGTTCTTCATCTTATATTTGGTCTATGAACTCCGGTCTTTCAACAGCAATTACCTACATATCTTGGAAGGTTGGACTTATTGGGATGTTTGAATTGTTTGTTGAATCACTAGTTAGACAACTTCAGAATAATTTTCTATATAAGTTTAGAACCCATATGATTTCTGCTTAATGTCTTCTCCTCGTTGGTGCATGTCATAAATAATTCCGGATATTTTTTCTGACACCATTGAACAGCAATGTCAAAGTCTTTTTTACATGTCTTGATCATAAATGAATGCTGTTATATGATGTTTGAATGGTGCTTCAATCAACTTAACTTGTTCATCTTGGAGAGAGGAATGACATTATGTGTATTTTTAACTGAATTGCTTAGTGGTCATCATGTTTTCTTTTCAGATTTTTTTTTGGATCCAACTGAGGCTAATAATTATG

>*MaDGK2*(Ma03_g02590) promoter

GGAAATATTCCCGGGTTGACCTGTACTCTCATGTGCCAGCTATAACTACACGGCACGAGCCAGCTCAGGCTCTTTCAGACGACCACGTCACCCGAGAAAATTACTCGGGTTGATTCGATCAAGCTCGGGAAAACAAAAGACCATCCGCTGCTTTCTTTTACGGAGTCGCCGACCCCGCTTGACCCGTTAAGAAAAAGGACGCAAACGTCCCGAGCACGCTGTACGCGCACATCGAATGCAATGTGGTGCGATGATGATATAAAGCAGTCGTCGGCCTCGATGGCCACCCCGCTTTCCTCCCGTTGCGGGTTCCCTCTCTTTCCCTCTCCTCCTCTTCTTCTCCTTTGCTTCGCCTCCAAGACAGATTCGACCGATAAATCGTATAAAAGGAGAAGAATTGCGGGGTTTGGATTTGGTTTTTGGTCGTACGTATCTCATCTCTTCCTCTTCGTTTGCAGTTTGTATTTCCGGTAATGTGGGATCGACCGATAGGGTTTTCCTTTTCCCTTTTCACTTTCCCCATTCTTTCTCGATTTGAGCTTACAAGGGTCCCGATCTGAAGAGGGATTTGCAGCCGTGGTGTCGGGTCGTGCAATTGGCTTCATGATTCTTGAATCTCTGTTGGGATTTTTGTTCTTTCTGTTGGATTTAGATTGGTTTCCTTGGGGATTCGTTGTTGATCAGTGCTATTCTGTATCGATTTGGGCTTCTGAGTTACTTGTCGTTTCTGTGATTTTTCTCGTGATTAAGCGTCTCAGGGCGGAAGTCTTTTTACATGAAATTGCTTACATTAAGCTTCGGTAGCTTAAAATTGATCTGCCTGTCGGAATTATGGCAAAAGTTCTCCACCTTCGACGTTGTCTGGGAACCCAAATCTTTAAGAACTACACAAGTTCCTGGAAGTTTGGACCTTTTAACCACTAGTCCGGCTATTTCACCATCATTTTCTATTATATATGTTAAGCAACCATATATGTTTTAAGGGTGCAAGTATAGACATTGAATTTTGTTAAGTTTCTTCTCCATGCTGGTGTATAATTTTCTTTACTATCGTTGTCTTATTGAATTAAAGTTTTCTTACAGTAGGTCAGAGTGCATCATATTACAGTAATATGCCACTTCTGGATGTTTTTTGCTGATGCCATTGAACAGCAACAACAAAATCTTTTACATGTCTTGATCTAAATGAATCTTGTTATCTTATGTTCATCTGGCAGTTCATTGAATTTACTTGTGTTTGGAAGGAGTAATGGTATTTTTGTACTTTAATTAAAATTCTCAGTGGTGATTGGGTTCTCTTTTACAGGTTTTCTTGGATCTTCAGAAACCATTTTCTTGCTAGAGTTATTTCCCTCTTTTGACTATGACCTGATGAGTGGAGCAACTGAGGCTAATAATG

>*MaDGK3*(Ma06_g05490) promoter

TCGATTATTGCCTCAAGACAGCACGTAAAGAATCACAAGCCTTGCCACAATAATTTCTCACATAGGCTGATATGGATGACAACTTAAATAATAATAATAATTAAAATTTTGATTATTGTATATTATAACTATTGAAAGTGAGCATTCCTTCTATTGATTCTTAAAGTACGCTTAAGATTTATGTAATTCAAATCAACAATCAAAGATCAGATTAAGATTTCAAAACATAGTATTTTTGATTATTTTGATGATCCACAATAAAAGCAATTCAAAAATTGTTAAATATAGGACCATAAAAATCAACCCAATCGATGATCAAAAGAATTTAGTTTGAAAGAAATACTAAAGTAGATTGATTATTTTTTTTAAAGTTTTATTTTCCAAAAAGAAAAATACATATTTATTTGATAATATGGATGAATATGTTAAACAATTATATCTTTGTATTATAAAATATTTTTTTAAATACAAACAGTTATGTTAAAATAAATATTTATATAAAAAAATAAAATAGAGCTGAATGACGTGGATAATATATATAGTTTGGTCCCCCAATAATTTCCACCTATATCATATTACTAATTTATGAAAGGAAAAGAGATTAACCATAAGCTTTTAGGGTTTATAATTGATTTAGAGGGATAATTTTGTTTTTCCTAATTTTATAATAATTCAGCGATTAATAGCTGTATATATTTCTGACTCTACATCGAGGAGTCAAATGTGGGGTCCGCAACTTCAAAGAGTCAAATGTGGGGTCCATCCACTACGGCGGTAAACGTTCGGGTAGGTTGAACAGGGTAAAGAAAATAGTACTTTGTTTGTCAACGGCTGTGGTCTGCGTAATATATTTAATTCAAATAAAGCAATTAATATATTTAATTCCTCCCCTTCCCGCGCTCGTCGCAGTTGTCGTGTCATATATTTAATTCAAATAAAGCAAATGGGAAGACAAAATAAAAAGGGTAAGAATAAATAAACTATTATGAATTCGAAAAAGAAGGATGCCGAAGGCTCGGTCCGTCGACCCTTTTGCCAGAGTTATTCCTTCCTCTTCCTCTCGGCTTCGTGTGCTTCGCTTTCTTGGGGAAGACGGGACGCGAGTGCGTAAATAGTGGCATGGAGCGAGCGAAAGGTAGCGATG

>*MaPLC1*(Ma02_g20790) promoter

GATGCATTTGGCAGCTGGAAGAGTGGAATTGCCATCAGCAGAACGGTGCAAACCATTATATTCCCAAATCGAGGAGCATCCCACTGAATGTTTTGAGGAAGAGCCGTGTAAGGTTAGCTACAATGCTTTCGATTATTTATGTTTTCTAATTATTTTCCGGGATGATTTTTCATAATAAAAAGAAAGCCTTTCTTTTTAGGCTTTTCCTGAAAGATGCCCCCTTTTTCAGATTTTCCTAACAGTATTTTTTTTTTTTTGTCTTTGTCTAATTCTTAATATACTCTTGACTTGACTTGATAGTTAGTCATTTATTTCATTTCTTTCTCTTTCTCTTTCTTTTTCTATCGAGGGATTTTTTTTATATTATTCAAAACACTTTTAACTCCATAAAAAATATTGAGGTTTTAGTGTTTTTAAAAATATAAAATATATTTTTTTTTGTATAGGTTCATAACAACACTCTAACTCATGTAAAAGTCAAATTATTTTAATATTTATTGTTTTAAAAGAAAGAACCATACTTATTTGAAATGGAACAAGGTTTATAAATATTTTCTTAATATGTTTTGAGTTCATTTCTTAATATATTTAGGAACATGAGCAATGTTCAGACTCAAAATATATTAGGGGAGTACTCAAATATATGGTTCTCAATTTAAGAAAATAAAAATTCAAATAATTTGATTTCTATATCAGTTATATAGTATTTCTATATACTTGAAATTAGACAAATAAAAAAAAGTAAAGCCTTTTCCAAGAAATCACCTTTCGGTATTCGTTAGATTGATTTATTTGGTAATCATGCTCGAACTTAGCTTATTTATAAAAGCAATGAAATCTCAAATTGGTTTACTAATCTCGACGAATAAATCCTTAACTCGTCAACGACTCGACTCGACTCGACTCGTGTCGGAGACTGTCGCCCACCGCCGAATCATTACTCTCACCAATGAGAAAGCTACTTATAGTCATTTTGGCTTTTCTATTTCCGGGCTTTCTTTCTGGATTACGGATCAAACAGGATAATTATGGATGTCTTACGACTAATCCACAATTCCATTGGTAAGGATAACTGGGTCCTACATTTCATGGTCCCACAATTCCCGGCTTTTATTCCTCGATTATAACTAAAATAGGAGGATAATAATAATAATAATAATAATAATAATAATAATAATAATAATAATAATANNNNNNNNNNNNNNNNNNNNATTATTATTTCTATTACTATTATATTATAAATTCCGAAATAGAAGCTTCGAACGGCCTCCTGCCGCGGAAGCAGCAGCGTTTCGACGGTTGGGAACGCGTTCCAACCTCGATGCCTGTCTCTGTCTGTCTGTCTCTCTCTCTCTCTCTCGCTCGCTTCTCTTTTCGGCCCCCTCGAGAACGCGAAGACAAGGAGGAAGGCCGCCGGGGGGAAGGAGGAAGGGGAGAGGGAGGAGGTGGAATCGCGGAATG

>*MaPLC2*(Ma01_g22520) promoter

GGAGTCTCAAGTGAGAATCGCATGACGCTCGTTTGATGACATCAAAGATGATATATATATACTCTTCCATGCTTTTTCTCCACGAGACACACACTTATGCTTTATTAATTCTCGGCATCAACTTCTCTTGTCCCCCATTTATATGCTTCTTCTTGCCAAAGCCTCATATAAAGAAATACAAGTTGGATCTTTACCTACCTATTTGTCTATCTCATGCAATTAACTTCAATAATAATAATAATAATAATAATGATGATGATGATGATATCATAAATTCTTAATTTTTATCATCATTATCATCATCATCAACAATGAGATACGTAAAATTAATTATCGATTTCATCTTTATAATATATATTTTTTTTCTTTCTCAATTTATGATCTTTAATTATTGACAAAATAAATCATCTGTTTAAAAAGCATTGTCATGACATAATAATAATAATATAATTTGCATCATGAATTCAATGAGATAAAGAGTGGGGGAATCGAGTTCTAAAGGTGGAGCCAATTAGAATGACGGGGCATCGCAAAGTGAGGAAGGGCACGCCTAACTCCAGTCGCATCCACGGCCGTCCAAGCGAAGAGATCAGTATGTGATTGTTATGATTTGCGGTGTCGATTGGAATTCTTCGTCTCGTGGGAGCAGCGGCGAATCGTATTCCCAACCACACACCATCAGAAACTTCTCAGGTGTCTGTGTGTCGGTCAAAAAGTGGACTCACTAACAGCTGACAGAAAACAACACAATAAGAAAATAATAATTAATTGTAGCAAAAAGCTTAAACGATGAAAAATGGAAATAGAAACTGTGCCCAAACAAAACATATAATAAATAAAAAGCCTATTCAAAAAATATTATAAACCTATACAAAACATAATATGATAAGAAATATTTAATACAAACTAATTTATGATAAGTTAAATAGTATTTCTCAAAAGGGATATAATGTTTTTGACACATCGATCGATAGATATATTATGAGCCGATGAGAATCTGCTTTTGAAATATCCTATCAAAACACTGATACTGATAGCAGAAACAAAAATGGGACGTAAAACTAAATTCCAACAGTACCGACCGTCCTCCGCAGCTATAAAGAGAAGGCGGTGGTGAAAACGGAGGGGCGGACAACCGTTTAAACTTACCCACGCAAAGAGAGAGAGAGAGGGAGGGAGGAAGGGGGACGCCGGCTGACGGCGCGGAGCGATG

>*MaNAC25*(Ma06_g33960) promoter

TGCTATGTTGCTGGAGTTGTGTGACATGGTCAATCTACTGTAATAAATATATATATATATATAGTTTGCATCTATTACGATGGCCTGTTGATAATATATATATGATGGATTGATTCAGTGATTGATGCTCGTCCTTCTAATCGAAGCCTCCTAATATTAGCCATCTATGGGCTTCGAAATTGACACTGTATTAACGATCTATAATCTTAAGCCAAGATGAAAGATTATGTTAAATCAACGATAATTAAGTTGAAACTTGTGTTATTATTATTGTAGTACAAAGATAATGAGCACATGACTCATCCAATAATGCGTCTCCATGGCATCATTTTCGATCATTTATGGAGGTGTGTTACTCAACTCCCTTTTCCCATTATCCAGTCAGTGAAACTCTCTCCGAGTCACATCACCACACCTTCCACGACGTAAAGCAACAGGTGACCAACGAAGGAGTCGGCGACTTCCTTCCTTCCTCTTAAGGGCCCCACGCGGTTTCTTCTCCTTCTGTAATTTTCTAGTGCTAATCGCTCAGTAACACACATGGTAGGGTGGCGTAGCGATCGTACACGTCGGAGGCCTCCTCACTTCCGGCTCAGCATGGGGAAGGTGGCAAGGGACCCATGGCGGGGTTGTCAAGGCTTGCATGGCGAATGGGATGATGACTCCAAGCTCCGAACAAGGCTTGCATGAACCCTCACCCGTCTGCTCTTTGACTCACGGTGATAACTTCCGCACCATTATTGCCTTCACAAGATCAGTGTGCCAGTCCACGGCACGGACCAGCTTCGACAAATTTATCCTTGCAAGTGGCTGAGTCTACACGCAGAAGAAGACAACCAGTGGAATGCCACATGGTTATGTTGCCGTCTGTCGTTCAGCAGTGAATCCATCCTGTCGATATAGCTCGGGATGAAAGCTAAGGTTGGTAGAATCTCAGTACAACATAAGGTAATTTGGGCAGTAAAAACAACCAAGTGGTGCAGCGTTTGAATATAGATTGGAGGCTTCTCCTACAAGTGACTTGCGCAGCACGTATGTTAGGCTTTTAGGAACCGAACCCCGCTATTTATGTGGATTCTCTCGCTTCCATACATCCTCTACTTTCTCAGGTCCACACAACACGCCTTGGTACACTTCTCCTTTCCATCTCCTGCTTTGTTCTCCTTTCTTGGGTCTCCAACCTGGTATTGGATGCATATGTTTCACGCCTTCTTTGCTTTGTGTCTAGAGGTGCAACCATG

>*MaNAC28*(Ma09_g09630) promoter

CTTTGACCACACTTAGATGCGGTGTCCATCCTTTGCTAAAACCTATAAGGATGATGAAGACACAAGATTCACAGGCATGAACGAAATTAGATAGAGTTGGCCATTCATTCCCATTTATTTCAGGCAATAGGTGCCTTCAAGTGAGTACACATATGATTCACAGGCATGAACAACGTCGCAACGCACTTTAGTCTTCGCAAGTGGTCGAAGATCCATTTTTGGCACTCCCATTTCTTTAATGCAAGTAAAATTATAATGATATATAGTTTAGTAATAACATTTTCCCTCAAGAAAATAATAGCAGTAGATAATTCAGAACGAGCCGCTGTGCTGGGATTGCTATAAGAGATTGGAATCGAGAATAGGGAAAAATGTTTGATATCATGCAGTATGACACTATATATTTGTATTGATTTCTTTCTCCCACTTGTAGTAGATTGTTCATGGCATGCTCACCCTTGCAGAAGTGATGGAAAGAACTGGCGTGCATGCGTGTTGATCTGCCATGAACCTTGCCATTCGCTCACTCGATCGTCTCTTCATCTTTCATCAGCCCACCTCATGGGAAAGGAACAGATTCCCAGCAGACTTTGAGGAAAGCATGGGTTGGAACTCTTTGTATTGTTCCTGCATTCCTATGTCACCTGGAACGATTCCACGCACGTTAAACAAAAAGAAGAGGGTGAAATGACTAGTAGATCAATGGCTGGAGCTTCCGCTGCATGCAAGTTTACACTCGTCATCCTGTCAATAGCCACAGACACTCGACGCCCGAGGACGGACTTTGTCATAGATCAGATGGTTGGATTCTTATCATCTGAAGACAAAGACCAAGCTTGATGGTGATATATTTGACATACATGACAGTGCAGATCCGATGTCGAAGAAGTCAAACAGAAAGAAATGTAGAGAGAGAGAGAGAGAGAGAGAGGTCAAAGGTGATGACTTGACTGAGGCTACTGACTGCCACCTTTGGTGACTCCCGCTGGTTCTATATATCTTCGGCTTCACATTTATACGCTCACCAAGAGGGTTCTGGTAAGAGTCTCTTTGCTTGCAAACACCTGAAACTTTGCTACATGAACAAAGAGCCAATCGTAGACAAGGATAAGAAGGGGGAGTAGAAGAAAGGAAGAATAATCATG
